# Comparative Systems Biology Reveals Allelic Variation Modulating Tocochromanol Profiles in Barley (*Hordeum vulgare* L.)

**DOI:** 10.1371/journal.pone.0096276

**Published:** 2014-05-12

**Authors:** Rebekah E. Oliver, Emir Islamovic, Donald E. Obert, Mitchell L. Wise, Lauri L. Herrin, An Hang, Stephen A. Harrison, Amir Ibrahim, Juliet M. Marshall, Kelci J. Miclaus, Gerard R. Lazo, Gongshe Hu, Eric W. Jackson

**Affiliations:** 1 Department of Plant Sciences, North Dakota State University, Fargo, North Dakota, United States of America, formerly USDA-ARS, Small Grains and Potato Germplasm Research Unit, Aberdeen, Idaho, United States of America; 2 USDA-ARS, Small Grains and Potato Germplasm Research Unit, Aberdeen, Idaho, United States of America; 3 Limagrain Cereal Seeds, Battle Ground, Indiana, United States of America, formerly USDA-ARS, Small Grains and Potato Germplasm Research Unit, Aberdeen, Idaho, United States of America; 4 USDA-ARS, Cereal Crops Research Unit, Madison, Wisconsin, United States of America; 5 School of Plant, Environmental and Soil Sciences, Louisiana State University, Baton Rouge, Louisiana, United States of America; 6 Department of Soil and Crop Sciences, Texas A&M University, College Station, Texas, United States of America; 7 Department of Plant, Soil, and Entomological Sciences, University of Idaho Research and Extension, Idaho Falls, Idaho, United States of America; 8 SAS Institute Inc., JMP Genomics Development, Cary, North Carolina, United States of America; 9 USDA-ARS, Western Regional Research Center, Genomics and Gene Discovery, Albany, California, United States of America; 10 Crop Biosciences, General Mill, Inc., Kannapolis, North Carolina, United States of America, formerly USDA-ARS, Small Grains and Potato Germplasm Research Unit, Aberdeen, Idaho, United States of America; Universidade de Sao Paulo, Brazil

## Abstract

Tocochromanols are recognized for nutritional content, plant stress response, and seed longevity. Here we present a systems biological approach to characterize and develop predictive assays for genes affecting tocochromanol variation in barley. Major QTL, detected in three regions of a SNP linkage map, affected multiple tocochromanol forms. Candidate genes were identified through barley/rice orthology and sequenced in genotypes with disparate tocochromanol profiles. Gene-specific markers, designed based on observed polymorphism, mapped to the originating QTL, increasing R^2^ values at the respective loci. Polymorphism within promoter regions corresponded to motifs known to influence gene expression. Quantitative PCR analysis revealed a trend of increased expression in tissues grown at cold temperatures. These results demonstrate utility of a novel method for rapid gene identification and characterization, and provide a resource for efficient development of barley lines with improved tocochromanol profiles.

## Introduction

Tocochromanols, collectively known as vitamin E, are synthesized in chloroplast membranes of plants and certain algae and bacteria [1]. A primary role of these compounds is to protect lipids in photosynthetic membranes and seeds against reactive oxygen species. Additionally, tocochromanols contribute to membrane function and integrity, electron transport, and cell signaling [2]–[5]. These functions have significance in normal plant growth and development, plant stress tolerance, and seed longevity [6]. Vitamin E is also influential in human health, providing antioxidant protection [7] and contributing to reductions in cardiovascular disease [8], cholesterol [9], and certain forms of cancer [10], [11].

The various tocochromanols have significant yet diverse biological activity. To date, a large proportion of vitamin E research (99.2%) has been centered on the tocopherol forms [12], particularly α-tocopherol. This is primarily due to early reports showing retention and distribution of α-tocopherol to be 80% greater than all other tocochromanols in humans and mice [13]. Unfortunately, reports that tocotrienols were more potent oxidative protectors [14], [15] did not result in research trends that would give equal emphasis to potency and concentration. Recent studies have shown that tocotrienol bioactivity differs from that of tocopherols. Nanomolar concentrations of α-tocotrienol have been shown to reduce neurodegeneration [16], [17], while tocotrienol compounds have been shown to reduce cholesterol [18], [19] and oxidative protein damage [20], and suppress human breast cancer [21].

**Figure 1:**
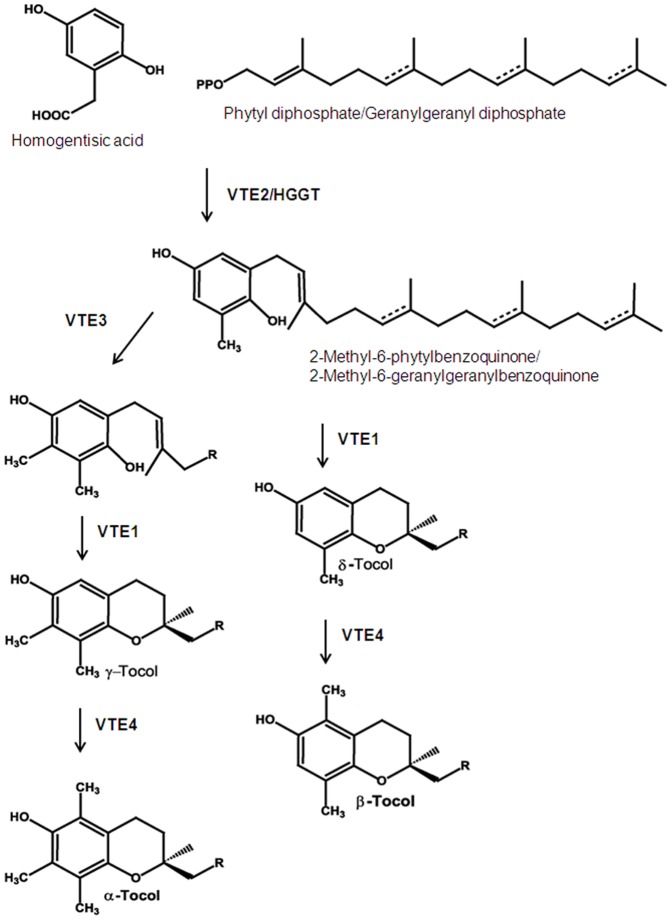
Tocochromanol biosynthesis. Dotted lines represent double bonds in the tocotrienol prenyl side chain. Dual labels correspond to tocopherol and tocotrienol, respectively.

Structurally, the tocochromanols consist of a polar chromanol ring and hydrophobic prenyl side chain (**Fig. 1**). Classification is based on chemical variations, with tocochromanols divided into tocopherols and tocotrienols based on the degree of saturation in the prenyl tail. Each tocochromanol type is further broken down into four forms (α, β, γ, and δ) based on the number and position of methyl groups on the chromanol ring [1], [22], [23].

Biosynthesis of tocochromanols involves two pathways. Homogentisic acid, the aromatic ring common to tocopherols and tocotrienols, is produced by hydroxyphenylpyruvate dioxygenase (*HPPD*), from products of the shikimate pathway [22], [23]. The methylerythritol phosphate pathway produces the prenyl side chains: threefold-unsaturated geranylgeranyldiphosphate for tocotrienols, and fully-saturated phytyldiphosphate for tocopherols [22], [23]. Partitioning of tocopherols and tocotrienols occurs during condensation of the chromanol ring and side chain, with preferential addition of the side chains by different enzymes. Homogentisate phytyltransferase (*VTE*2) primarily produces the tocopherol precursor, while homogentisate geranylgeranyltransferase (*HGGT*) primarily produces the tocotrienol precursor [22]–[24].

Remaining biosynthetic reactions are analogous between tocopherols and tocotrienols. Tocopherol cyclase (*VTE*1) closes the second ring in the head group and functions in synthesis of all four forms [1], [22], [23]. Form differentiation is caused by two methyltransferase enzymes: *VTE*3 methylation produces the γ form, which is otherwise identical to δ; and *VTE*4 methylation converts γ to α, and δ to β. Thus, *VTE*3 segregates γ and α forms from δ and β, and *VTE*4 segregates α and β forms from γ and δ (**Fig. 1**).

Small grains, which have high levels of the powerful and unique tocotrienol forms [24]–[28], are an excellent model for tocochromanol study. Although loci influencing tocochromanol biosynthesis have been identified on rice, maize, and oat chromosomes [29]–[32], utility of these QTL is limited by the breadth of the associated genetic region, the requirement for complex statistical models, and difficulty in merging QTL positions with sequence data to identify candidate genes [33]–[35]. Despite the potential impact of high-vitamin E barley, and the significance of this species as a model for more complex cereal grains, fundamental studies of barley tocochromanol genes have not been published.

Objectives of this research were to quantify tocochromanol accumulation in a population of Falcon x Azhul (FA) barley RILs, characterize candidate genes causing tocochromanol variation using a comparative systems biological approach, and develop predictive assays for novel alleles modulating tocochromanol levels and ratios in barley grain. Here we present genomic sequence data for tocochromanol synthesis genes in wild-type and mutant barley cultivars, correlating differences in tocochromanol profiles with sequence variation in genic and regulatory regions. We also present a genomic-based method for correlating significant genetic loci with the underlying operative gene. Application of this technique could augment the relevancy of the extensive but underutilized QTL data throughout the plant genetics literature. Progress in dissection of the genes identified in this study will facilitate development of barley cultivars with high total tocochromanol levels and desirable compound ratios, with applications for comparative human health studies and subsequent varietal release.

## Results

### Tocochromanol Analysis

Chemical analysis of the FA barley population detected all eight tocochromanol forms, with relative abundance of the four forms similar in tocopherols (T) and tocotrienols (T3) and total concentrations substantially higher in T3. Concentrations of α forms were highest, followed by γ, β, and δ. The δT form, although detected in 95.2% of samples, was present at low concentrations, with greater variance between replications. Falcon had higher levels of αT than Azhul or either check, although differences were not significant across all environments (**Table 1**). Tocopherol differences were less pronounced for the remaining three forms, although Azhul tended to have higher concentrations of γ and δ. Azhul accumulated higher levels of all T3 forms, particularly αT3.

**Table 1:** Tocochromanol means of Falcon and Azhul parents and two barley checks grown over four location years.

		Tocopherol				Tocotrienol				
Environment	Genotype	α^a^	β	γ	δ	α		β	γ	δ
AB^b^ 2008	Baronesse	10.75 B	1.30 A	4.31 A	0.75 A	27.16 C		9.34 A	7.93 A	1.45 A
	CDC Alamo	10.51 B	1.12 A	2.87 AB	0.41 B	32.06 B		4.52 B	7.52 A	0.96 B
	Falcon	11.93 A	1.11 A	1.60 B	0.14 C	29.00 BC		5.49 B	5.62 B	0.84 B
	Azhul	10.47 B	1.06 A	2.29 B	0.23 BC	35.33 A		5.54 B	8.37 A	1.47 A
	*Mean*	10.91***	1.15*	2.77***	0.38**	30.89** +		6.22***	7.36***	1.18***
AB 2009	Baronesse	12.90 B	1.44 A	8.62 A	1.44 A	35.58 B		16.37 A	9.23 B	1.94 B
	CDC Alamo	13.09 AB	0.92 B	5.06 B	0.60 B	37.41 B		5.53 D	10.02 B	1.03 C
	Falcon	13.99 A	1.39 A	2.36 C	0.55 B	35.30 B		9.15 C	8.16 C	1.27 C
	Azhul	12.71 B	1.22 A	4.14 B	0.76 B	48.03 A		13.10 B	12.03 A	2.49 A
	*Mean*	13.17*	1.24*	5.04*	0.84*	39.08*		11.04*	10.04*	1.68*
AB 2008–2009	FA RIL (H^2^ d)	12.00 (*35*)	1.23 (*55*)	2.82 (*51*)	0.47 (*47*)	36.02 (*45*)		9.41 (*54*)	8.43 (*55*)	1.54 (*61*)
TE^c^ 2008	Baronesse	10.45 A	0.92 A	3.48 A	0.28 A	26.75 AB		9.37 A	9.33 A	1.50 A
	CDC Alamo	10.15 A	0.86 A	3.17 A	0.28 A	28.61 AB		4.16 B	8.10 A	0.89 AB
	Falcon	11.23 A	0.99 A	1.67 C	0.10 B	26.94 B		6.08 B	6.86 A	1.04 AB
	Azhul	10.41 A	0.99 A	2.59 B	0.16 AB	33.04 A		5.13 B	8.06 A	0.92 B
	*Mean*	10.56***	0.94**	2.73***	0.21***	28.83***		6.19***	8.09** +	1.09***
TE 2009	Baronesse	11.92 AB	0.96 A	6.40 A	0.93 A	30.54 BC		13.73 A	9.05 A	1.67 A
	CDC Alamo	12.21 AB	0.71 B	4.07 B	0.39 B	31.69 B		4.78 C	9.12 A	1.10 B
	Falcon	12.31 A	0.74 B	1.59 D	0.26 B	28.67 C		7.13 B	6.56 B	1.01 B
	Azhul	11.36 B	0.77 AB	2.34 C	0.23 B	37.00 A		7.72 B	9.89 A	1.80 A
	*Mean*	11.95**	0.80***	3.60**	0.45**	31.98**		8.34**	8.66**	1.39**
TE 2008–2009	FA RIL (H^2^)	11.11 (*41*)	0.86 (*48*)	2.42 (*58*)	0.25 (*44*)	30.04 (*57*)		*7.22 (*63*)*	7.58 (*61*)	1.28 (*59*)

Means and heritability estimates were calculated for Falcon x Azhul (FA) RILs using combined years at each location.

a Concentrations given in µg/g. Values followed by the same letter or number of asterisks are not significantly different within a column (p<0.05). For means of genotypes within an form, a single asterisk is equivalent to A, two asterisks to B, and three asterisks to C.

+ indicates an intermediate value.

b Irrigated field trial in Aberdeen.

c Non-irrigated field trial at Tetonia.

d Broad sense heritability calculated as genotype variance divided by cumulative variance including error.

Analysis of variance indicated significant influence of location and year (**Table S1**). With the exception of βT in Tetonia, mean levels of each form were higher in 2009 than in 2008 (**Table 1**). For both years, concentrations of all forms were higher in Aberdeen than in Tetonia (**Table 1**). Within the FA RILs, broad sense heritability estimates ranged from 35 to 61 in Aberdeen, and from 41 to 63 in Tetonia, with generally higher heritability for T3.

### Comparative Genomics Candidate Gene Identification

Molecular genetic dissection of tocochromanol accumulation in barley grain was achieved through general pathway elucidation using model species, followed by identification of genomic areas associated with acute phenotypic measurements of each bioactive compound. This information was then used to resolve candidate genes based on comparisons with annotated genes from related species, allowing candidate gene validation using wild-type and mutant genotypes.

**Figure 2:**
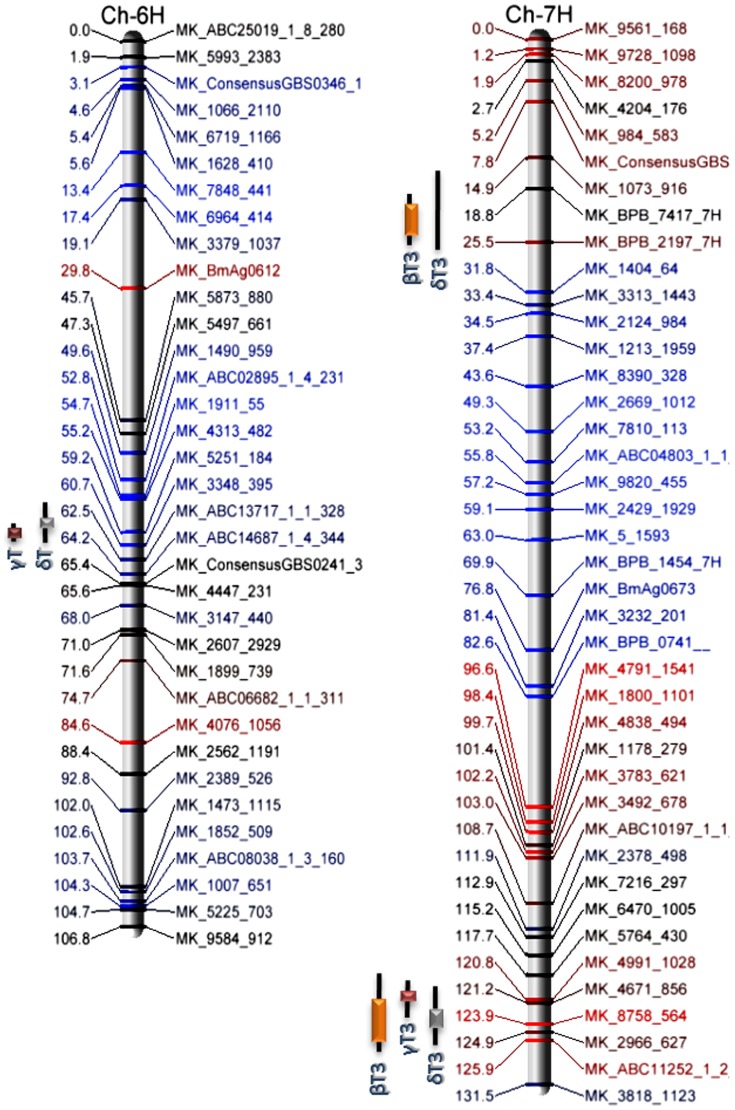
Major QTL regions influencing tocochromanol biosynthesis in the barley Falcon x Azhul RIL population. QTL common to all environments are indicated by a colored bar. Black extensions indicate regions affected by at least one environment. Marker names and map positions are colored by additive effect of parental alleles. Blue indicates a greater influence of the Falcon allele; red a greater influence of Azhul.

Genomic regions affecting phenotypes in at least three environments were detected on two barley chromosomes (**Table 2** and **Fig. 2**). Each region encoded enzymes for the production of multiple compounds of either T or T3: chromosome 6H affected γT and δT accumulation, and two loci on 7H affected βT3 and δT3, with the second also significant for γT3. Based on rice and barley orthology, 6H contained a *VTE*4 sequence while genomic regions of 7H contained sequence for *VTE*2 and 3-phosphoshikimate 1-carboxyvinyltransferase, which synthesizes a distant precursor of the tocochromanol ring structure (**Fig. S1**). Additional genomic regions affecting βT phenotypes from two environments were localized on 1H and 3H. Although non-significant, genomic regions were identified on 5H affecting βT and δT3, and on 6H affecting δT3. Barley-rice orthologous relationships showed the genomic region on 5H contained *VTE*3, and the region on 6H contained *VTE*1. Since a SNP marker was not mapped at the QTL peak on 5H, analysis of this locus was based on an additional sequence identified through the HarvEST database (**Fig. S1**). Of the two parental lines, Azhul appeared to have a stronger influence on T3 variation, predominating at both 7H QTL regions (**Fig. 2**). The Falcon allele appeared to have a greater influence on T accumulation, evidenced by the region on 6H.

**Table 2:** Tocochromanol quantitative trait locus analysis summary for Falcon x Azhul RILs across four location years.

					Original			With VTE4			With HGGT		
Chrom.	Trait^a^	Environ.^b^	QTL marker^c^	QTL peak (interval)^d^	LOD^e^	Add.^f^	R^2g^	LOD	Add.	R^2^	LOD	Add.	R^2^
6H	γT	AB 2008	MK_4313-482	57.2 (56.4, 58.0)	12.43	0.51	0.31	17.42	0.65	0.50	12.43	0.51	0.31
		AB 2009	MK_4313-482	57.2 (56.9, 60.9)	15.82	0.90	0.37	14.11	0.95	0.41	15.57	0.90	0.37
		TE 2008	MK_4313-482	57.2 (55.3, 58.6)	7.76	0.37	0.21	13.50	0.53	0.45	7.76	0.37	0.21
		TE 2009	MK_4313-482	57.2 (56.6, 58.2)	12.86	0.60	0.30	15.12	0.71	0.43	12.86	0.60	0.30
	δT	AB 2008	MK_4313-482	55.2 (55.1, 57.3)	11.30	0.09	0.26	12.64	0.11	0.37	11.00	0.09	0.25
		AB 2009	MK_4313-482	57.2 (56.4, 58.6)	8.69	0.16	0.22	9.06	0.17	0.26	8.69	0.16	0.22
		TE 2008	MK_1911-55	54.7 (53.7, 57.4)	5.98	0.03	0.14	6.58	0.04	0.23	5.98	0.03	0.14
		TE 2009	MK_4313-482	57.2 (54.2, 57.8)	8.94	0.09	0.20	9.31	0.09	0.19	8.89	0.09	0.20
7H	βT3	AB 2008	BPB-7417_7H	22.8 (19.9, 23.9)	11.61	−0.95	0.36	12.42	−2.07	0.36	12.66	−1.88	0.34
		AB 2009	BPB-7417_7H	21.2 (19.7, 23.2)	18.19	−3.27	0.42	18.41	−3.33	0.43	21.23	−3.51	0.47
		TE 2008	BPB-7417_7H	20.9 (19.9, 23.4)	12.82	−2.03	0.36	13.12	−2.03	0.36	12.96	−1.99	0.35
		TE 2009	BPB-7417_7H	20.8 (19.0, 23.2)	17.11	−2.53	0.41	16.82	−2.42	0.38	17.79	−2.54	0.41
	δT3	AB 2008	BPB-7417_7H	20.8 (19.9, 23.8)	10.37	−0.40	0.29	10.57	−0.41	0.31	9.99	−0.33	0.24
		AB 2009	BPB-7417_7H	18.8 (16.4, 22.9)	11.90	−0.46	0.23	11.76	−0.46	0.23	14.37	−0.50	0.28
		TE 2008	MK_1073-916	16.9 (14.1, 18.1)	7.39	−0.32	0.22	8.19	−0.34	0.24	7.11	−0.31	0.20
		TE 2009	MK_1073-916	16.9 (15.9, 22.0)	12.81	−0.41	0.28	10.41	−0.34	0.22	11.61	−0.35	0.24
7H	βT3	AB 2008	MK_4671-856	123.2 (120.1, 126.3)	2.75	0.82	0.06	2.61	0.71	0.05	5.12	1.04	0.10
		AB 2009	MK_5764-430	119.7 (117.4, 125.7)	5.28	1.57	0.10	5.36	1.57	0.10	7.56	1.83	0.13
		TE 2009	MK_4671-856	123.2 (117.1, 126.0)	3.47	1.00	0.06	2.85	0.85	0.05	5.15	1.15	0.09
	γT3	AB 2008	MK_5764-430	119.7 (117.8, 120.4)	4.25	0.87	0.12	-	-	-	6.77	0.99	0.16
		AB 2009	MK_5764-430	119.7 (117.4, 120.4)	7.97	1.35	0.23	8.04	1.35	0.23	11.80	1.56	0.31
		TE 2008	MK_5764-430	117.7 (116.6, 124.4)	3.43	0.80	0.09	3.93	0.87	0.10	7.79	1.19	0.19
		TE 2009	MK_5764-430	119.7 (118.6, 120.6)	8.15	1.08	0.24	7.64	1.02	0.22	10.81	1.28	0.34
	δT3	AB 2008	MK_4671-856	123.2 (121.6, 124.7)	5.08	0.24	0.12	4.69	0.21	0.09	7.80	0.27	0.17
		AB 2009	MK_4671-856	123.2 (120.9, 123.4)	9.35	0.42	0.19	6.77	0.34	0.11	11.27	0.44	0.21
		TE 2009	MK_4671-856	123.2 (121.6, 124.9)	6.03	0.26	0.12	5.91	0.23	0.10	7.36	0.28	0.15

a Indicates the tocochromanol form. T – Tocopherol; T3 – Tocotrienol.

b AB indicates the irrigated field trial in Aberdeen; TE indicates the non-irrigated field trial in Tetonia.

c Flanking marker to the left of the QTL peak.

d Interval based on one LOD to each side of the peak. Peak and interval positions are in cM.

e QTL detection was based on a LOD threshold of 2.5 (1000 permutations and type I error of 5%).

f Total phenotypic variation explained by all QTL.

g Percentage of phenotypic variation accounted for by QTL.

### Gene sequence and expression analysis

**Figure 3:**
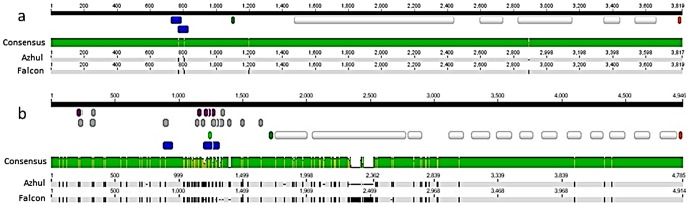
Sequence alignments for gene and promoter regions of VTE4 (A) and HGGT (B) in barley cultivars Falcon and Azhul. Polymorphisms are indicated by black interruptions within the gray bars, with narrow sections corresponding to indels. Annotations are indicated by colored bars above the consensus guide. Within the promoter region, blue indicates a promoter sequence, light green a TSS, purple a TATA or CAAT box, and gray all other motifs. The transcription start codon is indicated in green, and the stop codon in red. White bars indicate introns.

Genes corresponding to the two major QTL regions are substantially different in genetic structure and polymorphism level, but similar in gene expression profile. The full-length *VTE*4 gene comprised six exons and five introns, ranging in size from 84 to 952 bp (**Fig. 3a**). Approximately 54% of the total gene length is intronic. Polymorphism was limited to a 2-bp indel in the third intron, and a wobble base in the first exon of Falcon (**Table S2**). The nucleotide variation changes the amino acid, with the G variant encoding alanine and the T variant encoding serine. Ten clones, based on two different cloning events, were sequenced in an attempt to resolve the nucleotide identity at this position: five clones contained a G and five a T, with sequence identity proportional within each cloning event. Remaining sequence, including the intronic indel, was identical within these clones.

Sequence analysis of 1100 bp upstream of the *VTE*4 start codon revealed two sequence polymorphisms between Falcon and Azhul, both of which fell within promoter sequences (**Fig. 3** and **Table S2**). Approximately 18 additional types of motifs were annotated at approximately 90 sites within the sequenced promoter region, including motifs for response to low temperature, light, and plant stress (**Table 3** and **Table S3**).

**Table 3:** Motifs affected by promoter sequence polymorphism between Falcon and Azhul.

Adjacent gene	Bp upstream of ATG acpoisiont	strand	Motif function	Motif sequence	Sequence	
					Falcon	Azhul
*VTE*4	293	+	Promoter of transcription start	TATA	TACA	TATA
	328	+	Cis-acting element, transcription start	CCAAT	TCAAT	CCAAT
*HGGT*	381	+	Cis-acting element, low temperature response	CCGAAA	ACCGAAA	ACCCGAAA
	452	+	Promoter of transcription start	TATAAA	TATATG	TATAAA
	458	-	Promoter of transcription start	TATACA	TATACC	TATACA
	518	+	Light response element	AGAGATG	____ATG	AGAGATG
	534	+	Cis-acting element, endosperm expression	GTCAT	GTCGT	GTCAT
	579	+	Auxin-responsive element	AACGAC	AACCAC	AACGAC
	581	-	Cis-acting element, MeJA responsiveness	TGACG	TGACC	TGACG
	620	-	Cis-acting element, low temperature response	CCGAAA	CTGAAA	CCGAAA
	824	-	MYB binding site, drought-inducibility	TAACTG	TAACTGG	TAACTGT
	1006	+	Cis-acting element, light responsiveness	CACATGG	ACACATGG	GCACATGG

Given the similar function of *VTE*2 and *HGGT*, cloning of both genes was attempted. Amplification was not obtained for *VTE*2; however, *HGGT* amplified readily. The *HGGT* full-length gene contained 14 exons and 13 introns, with introns comprising approximately 60% of the total length and ranging in size from 75 to 715 bp in Falcon (**Fig. 3b**). Polymorphism was pronounced, tending to occur more frequently in introns and in the 5′ half of the gene. Even accounting for relative intron/exon lengths, intronic SNPs occurred at nearly twice the frequency of those in exons. All indels were within introns, including a region of approximately 150 bp in the second intron. *HGGT* is known for assembling T3; thus, two FA RILs (FA.41 and FA.117) with similar total T levels (13.82 and 14.10 µg/g) and disparate total T3 levels (35.51 and 69.49 µg/g) were also cloned and sequenced. Gene sequences were identical between FA.41 and Falcon, and between FA.117 and Azhul, correlating sequence data with low and high total T3 phenotypes.

**Figure 4:**
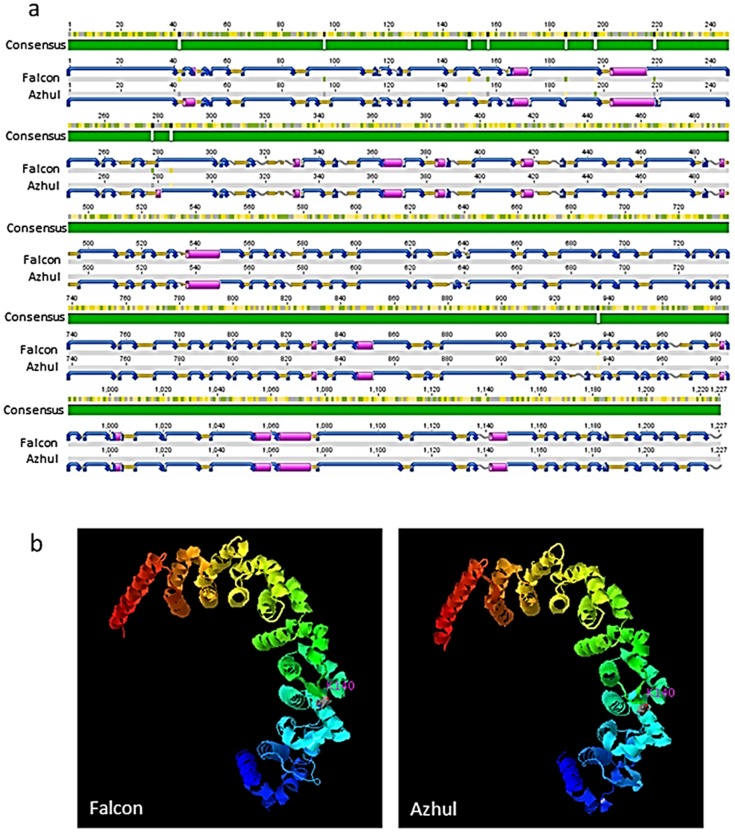
Predicted secondary structure (A) and three-dimensional structure (B) of HGGT proteins in barley cultivars Falcon and Azhul. Sequence polymorphism contributing to folding changes is indicated by the green consensus guide (A). Blue curved arrows represent turns, orange arrows represent beta strands, pink cylinders represent alpha helices, and gray wavy lines represent coils. Pink labels within ribbon diagrams correspond to the predicted active site.

Protein structure prediction was performed to understand the effects of allelic variation within *HGGT*. Of ten FA SNPs within the coding sequence, seven were silent and three caused an amino acid change affecting polarity or pH (**Table S4**). Although all SNPs sites were predicted to affect secondary protein structure (**Fig. 4a**), no significant three-dimensional structural or functional differences were predicted based on I-TASSER analysis of Falcon and Azhul protein sequences (**Fig. 4b**). The best-ranked enzyme classification scores were 0.851 for Falcon and 0.856 for Azhul, both matching peptide homolog 1dceA corresponding to the alpha subunit of geranylgeranyltransferase. The active site was predicted at residue 140. Of the remaining best five matches, only 1d8dA and 3dsxA were common to both allele peptides.

The lack of evidence for substantially altered protein structure in HGGT warranted further evaluation of the promoter region. Analysis revealed SNPs in motifs influencing low temperature response, light response, drought tolerance, auxin response, TATA and CAAT boxes, promoter sequences, including a transcription start site, and others (**Table 3** and **Table S3**). The sequenced promoter region included approximately 30 types of promoter motifs at approximately 100 sites (**Table S3**).

**Figure 5:**
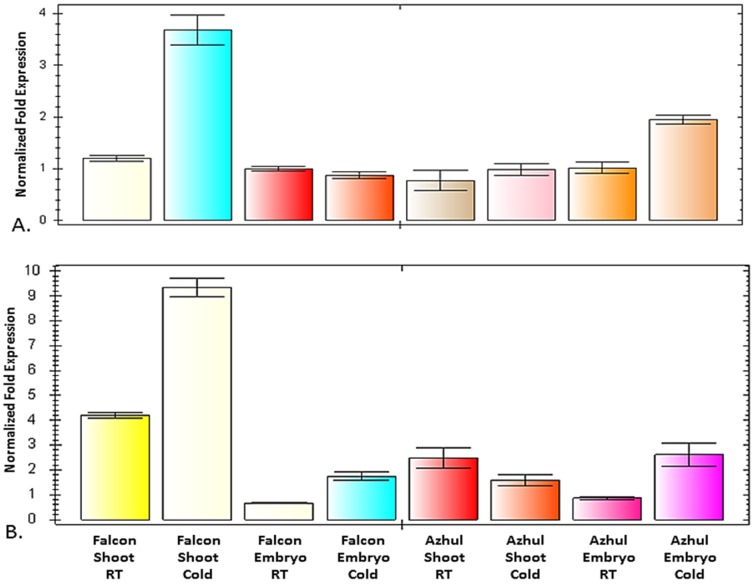
Quantitative PCR analysis of *VTE*4 (top) and *HGGT* (bottom) in Falcon and Azhul barley cultivars. Tissue samples used for cDNA preparation were taken from shoots and embryos grown at room temperature and at 4°C.

Both *VTE*4 and *HGGT* showed a trend of increased expression in response to cold temperatures (**Fig. 5**). Temperature-based expression differences were pronounced in samples based on Falcon shoot tissue, with an approximately threefold expression difference in *VTE*4, and more than a twofold difference in *HGGT*. Samples based on Azhul embryo tissue showed a similar response, with cold temperatures increasing expression twofold in *VTE*4 and nearly threefold in *HGGT*. Temperature did not cause a statistically important effect in *VTE*4 for Falcon embryo tissue or Azhul shoot tissue. However, samples based on Falcon embryos showed more than a twofold increase in *HGGT* expression in response to cold. Expression of *HGGT* in Azhul shoots appeared to decrease in response to cold, although the difference was not statistically significant.

### Predictive markers and validation of gene identification

Predictive PCR-based markers for were designed for*VTE*4 and *HGGT* genes (**Table S5**). The *VTE*4 marker amplified a 78-bp region containing the 2-bp indel in the third intron; the *HGGT* marker amplified a 53-bp region surrounding the penultimate SNP, in the eleventh exon (**Fig. 3**). Both markers generated a 1∶1 ratio when genotyped in the FA population (**Table S6**) and mapped to the respective QTL peaks when appended to the FA genetic linkage map (**Fig. 2**). Reiteration of marker-trait correlation, including predictive markers, increased the mean LOD score, additive variance, and R^2^ values at the respective loci (**Table 2**). Inclusion of the *VTE*4 marker increased the mean R^2^ value from 0.25 to 0.36; addition of the *HGGT* marker increased the mean R^2^ from 0.13 to 0.19. Mean values for additive variance increased from 0.34 to 0.41 for *VTE*4, and from 0.84 to 1.00 for *HGGT*. In addition, inclusion of the *HGGT* marker allowed detection of additional peaks at the *HGGT* locus, making the QTL significant for βT3, γT3, and δT3 in all four environments, and for αT3 in all environments except AB 2008.

## Discussion

Given the complexity of systems biology and the scope of plant genetics research, species-specific elucidation of even the major biochemical pathways is unrealistic; thus, a detailed and finely-curated set of biochemical pathways in Arabidopsis and other models has been critical to assessment of location, structure, and function in corresponding genes of organisms with larger and more complex genomes. Characterization of the tocochromanol pathway in Arabidopsis has accelerated elucidation of vitamin E production in soybean and other plants in which vitamin E is a key trait. However, the comparative genomics approach has not been extended to correlation of genes or sequence data within QTL. Here we demonstrate the application of biochemical and sequence data from model species to directly elucidate corresponding genes in more complex genomes.

Significant variation in field location and year indicate that seed tocochromanol content is impacted by environment. Variation between years appeared to be correlated to temperature and possibly water availability. Mean overall temperatures for both locations were approximately 1°C lower in 2009 than in 2008; thus, cooler conditions within the same location favored increased tocochromanol production. This conclusion concurs with previous reports. For example, environmental impacts on tocochromanol production were reported in a study of wheat, canola, sunflower, and soybean. Although individual field effects were not separated, growth chamber experiments verified a strong correlation between temperature and vitamin E content, with lower temperature causing increased accumulation [36]. Temperatures in Tetonia were colder than in Aberdeen; however, tocochromanol production was lower, possibly reflecting differences in water availability, cultivation practices, or saprophytic fungi or bacteria. Although not fully understood, the trend is consistent with previous data. Levels of αT and αT3, measured in an oat population grown at the same locations, were significantly lower in Tetonia [32]. Given the sequence variation in promoter motifs related to temperature and drought response, interaction of barley genotypes with environmental impacts is not surprising (**Table 1** and **Table 3**).

As in other barley tocochromanol studies [37], [38], αT and αT3 were the predominant forms, with αT3 being the more abundant. Within each tocochromanol type, concentrations of γ forms were approximately one-fourth as abundant, followed by β, and more distantly by δ forms. This indicates that γ and δ precursors are effectively converted to α and β forms in mature barley seed, reflecting activity of *VTE*4 (**Fig. 1**). Spatial and quantitative accumulation of the eight forms has been shown to vary during barley seed development, with tissue-specific form distribution, and relative form ratios shifting throughout early kernel formation [39]. Kinetics of tocochromanol forms suggests specific roles throughout seed development and germination, requiring precise control of individual genes.

In this study, major loci affected multiple forms, reflecting activity of genes at key intersections of the tocochromanol biosynthetic pathway. The barley 6H QTL influencing γT and δT corresponded to a rice region containing *VTE*4, which converts γ to α and δ to β (**Fig. 1** and **Fig. S1**), accounting for the forms mapped to this locus. Minimal FA sequence variation was observed within the *VTE*4 gene and its upstream regulatory region, although observed polymorphism affected significant regions, modifying two promoter motifs and causing an amino acid change. Based on *VTE*4 sequences of two FA RILs with divergent levels of γ and δ forms, it does not appear that the intronic indel plays a role in *VTE*4 activity. However, the indel is heritable, evidenced by the 1∶1 ratio produced when genotyping the FA population with the molecular marker based on this polymorphism (**Table S6**). Despite the sequence similarities, expression patterns of *VTE*4 differed between Falcon and Azhul, with cold temperatures inducing increased expression primarily in Falcon shoot tissue and Azhul embryo tissue. Since promoter sequence polymorphism affected regions involved in low-temperature response (**Table 3** and **Table S3**), these motifs may affect spatial expression of *VTE*4. Future work should be performed to explore this possibility, as well as to study additional factors affecting *VTE*4 gene expression such as methylation patterns and involvement of microRNA.

The QTL accounting for a majority of the phenotypic variance in this study were detected on chromosome 7H. Both regions influence accumulation of multiple tocotrienol forms, the more abundant tocochromanol in barley and some other small grains. The QTL affecting all four T3 forms corresponded to the rice chromosome 6 region containing *VTE*2 (**Fig. 2** and **Fig. S1**), a gene reported to preferentially add the phytyldiphosphate side chain to form tocopherols, with analogous formation of tocotrienols by *HGGT* [24]. Dicots appear to lack a prenyltransferase recognizing geranylgeranyl diphosphate, hence they produce little or no tocotrienols. Monocots, however, appear to express two distinct prenyltransferases, one favoring phytyldiphosphate as substrate, the other favoring genanylgeranyl diphosphate (although there is some overlap in substrate specificity with both enzymes)[24], [40]. In this study, a major QTL on chromsosome 7H corresponded with increased accumulation of all four tocotrienol forms. Barley SNP sequences within this QTL are syntenous with a rice chromosome 6 region containing *VTE*2 (OS06g44840), closely neighboring a region containing *HGGT* (OS06g43880). In this study, barley *HGGT* corresponded with the rice region containing *VTE*2, and a barley *VTE*2 locus was not identified, suggesting minimal activity of this enzyme. Based on the high level of barley/rice orthology, it is likely that the two genes are adjacent in barley as well as rice. This genetic proximity, coupled with the apparently dichotomous gene expression, suggests that transcriptional interference [41] may contribute to regulation of these genes. This could account for the increased proportion of tocotrienols in barley and other small grains, and the established predominance of one tocochromanol subgroup within a plant family [24]–[28], [42]. Promoter motifs identified in the regulatory regions of both genes were consistent with known gene functions. Both tocopherols and tocotrienols have been shown to provide photoprotection of chloroplast tissue, with biosynthesis increased under high light intensity [43]–[45]. The tocochromanols also serve to protect plant tissue from cold temperatures, oxidative stress, high salt environments, and other stress conditions [2]–[6], [43]. Light response elements were prevalent in both regulatory regions, occurring at three distinct sites in the promoter region of *VTE*4 and at approximately 21 sites in the upstream region of *HGGT* (**Table S2**). Motifs for low temperature response and anaerobic induction were also common to both promoter regions. Frequent Falcon/Azhul polymorphism affecting motifs within the upstream region of *HGGT* suggests that expression may be substantially altered by sequence changes within these binding sites. For example, a barley motif implicated in low temperature response [46] occurred at two polymorphic sites in the Falcon and Azhul *HGGT* promoter regions (**Table 3**). Previous studies [46] have demonstrated that the hexanucleotide sequence comprising this motif is the binding site for a single protein complex, and that mutation of the motif sequence reduces responsiveness of barley to low temperatures.

Taken together, these results suggest that barley tocochromanol accumulation is regulated by a complex and interdependent network of genes, providing at various times the forms required for seed development, plant growth, and stress response. Specificity of form control at different loci could enable selection for desired ratios of tocochromanols, enhancing seed storage longevity and nutritive profile. The identification and characterization of major genes affecting tocochromanol biosynthesis will facilitate such selection, allowing rapid development of barley lines with improved vitamin E content and novel form ratios. This in turn will provide plant resources for studying the effects of tocotrienols on human health, and may lead to a novel phytonutrient food source.

## Methods

### Plant material and field experiments

A population of 142 recombinant inbred lines (RILs) was derived from hybridization of six-row barley ‘Falcon’ (F) and ‘Azhul’ (A). Falcon is a hulless feed barley (PI591612) well adapted to Idaho; Azhul is a dwarf, hulless food barley derived from mutation breeding (released by the USDA-ARS and the Arizona Agricultural Experiment Station). The FA population was derived by single-seed descent to the F_6_ generation, and F_8_ seed was harvested from individual F_7_ plants to produce seed for each RIL.

Field trials were performed in a completely randomized block design and included three replications of F_6:8_ RILs and 6–12 replications each of Falcon, Azhul, and two-row barley checks ‘Baronesse’ and ‘CDC Alamo’ (industry leaders in feed and food types, respectively). Trials were conducted over two years (2008 and 2009) at two locations: irrigated at the USDA-ARS in Aberdeen, ID, and non-irrigated at the University of Idaho Tetonia Research and Extension Center, Tetonia, ID. Plots consisted of 1.2-meter rows with 0.4 m spacing between rows. Fertilizer and post-emergent herbicide was applied, and seed was combine harvested.

### Tocochromanol analysis

Barley seed was ground in a Retsch ZM-1 mill and extracted using a revised hot saponification and extraction method [47]. Briefly, 0.5 g freshly-ground sample was weighed into a 15-mL screw-cap glass test tube and mixed with 0.5 mL potassium hydroxide (600 g/L H_2_O), 0.5 mL ethanol (95%), 0.5 mL sodium chloride (10 g/L H_2_O), and 1.25 mL ethanolic pyrogallol (60 g/L EtOH). Solution was mixed with a glass rod. Tubes were then vortexed and saponified in a 70° water bath for 30 min., with vortexing every 10 min. Tubes were chilled in an ice bath for 15 min. The solution was extracted twice, once with 3.75 mL sodium chloride (10 g/L) and 3.75 mL n-hexane/ethyl acetate (9∶1), once with 3.75 mL n-hexane/ethyl acetate, and organic layers were evaporated in a speed vacuum. Dried residue was resuspended in 2 mL hexane. Chromatographic separation was performed with normal phase HPLC, using a Shimadzu (Kyoto, Japan) LC-6A pump, RF10AxL detector, and SCL-10Avp controller. Compounds were separated on a 5-µm 250×4.6 mm Grace Adsorbosil silica column using an isocratic mobile phase (hexane with 2% dioxane and 2% ethyl acetate) at a flow rate of 2 mL/min. Peaks were detected by fluorescence with excitation at 295 nm and emission at 330 nm. Identification of individual forms was based on retention time, and quantification was based on standard curves developed using authentic tocopherols (Metraya LLC, Pleasant Gap, PA). Tocotrienols, having essentially the same fluorescent properties as their corresponding tocopherols [48], were quantified with the same standard curves.

### QTL mapping

Genotyping and linkage analysis were performed according to Islamovic et al. [49]. Analysis of variance (ANOVA), means, and broad sense heritability estimates were calculated for each tocochromanol form using the JMP 9.0 software (SAS Institute, Cary, NC). Heritability within the RIL population was calculated by dividing genotypic variance by total variance, calculated from variance components estimates for genotype, location, and year in a full-factorial random effects model. Based on significant interactions within the model, each genotype, location, and year were treated as separate traits for QTL identification (**Table 1**).

QTL were detected with WinQTL Cartographer [50], using marker locus positions from an existing Falcon x Azhul SNP map [51]. Experiment-wise thresholds, determined through 1000 permutations of the data, were used to determine significant QTL [52]. Peaks were initially detected using single marker analysis, and QTL positions, LOD scores, and phenotypic variance were determined with composite interval mapping (CIM)[53], using stepwise regression, a window size of 10 cM, walk speed of 2 cM, and significance threshold of *P* = 0.05. Clustered peaks were counted as separate QTL if individual peaks spanned more than one LOD score.

### Orthology of barley QTL to rice sequence

Sequences from SNP markers within one LOD score of significant QTL peaks were used to identify syntenous rice regions, using the BLAST tool of the Rice Genome Annotation Project (http://rice.plantbiology.msu.edu). The Rice Genome Browser was then used to identify candidate rice genes within the region of the QTL peak. For barley regions with widely-spaced markers, additional SNPs within the QTL were identified using the HarvEST:Barley software version 1.77 (http://harvest.ucr.edu). Candidate genes likely to influence trait expression were identified based on map position and function.

### Sequencing and annotation of tocochromanol genes and regulatory regions

Primers for gene cloning were designed based on coding sequences of locus AK355075 for *VTE*4, locus AK366699 for *VTE*2, and locus AY222860 for *HGGT* (available in NCBI http://www.ncbi.nlm.nih.gov). Primers for promoter regions (contigs 254212 and 2207823 for *VTE*4, contig 1025503 for *HGGT*) were designed from sequences obtained from the morex_rcba database in ViroBLAST, Leibniz Institute of Plant Genetics and Crop Plant Research (IPK) (http://webblast.ipk-gatersleben.de/barley/viroblast.php). Primer sequences are listed in Table S5. PCR amplification was performed using the Phusion High-Fidelity PCR Kit (Finnzymes, #F-553S, distributed by New England BioLabs, Inc.). Reaction preparation and thermocycling were generally as recommended by the manufacturer, with 40 cycles of 10 s denaturation, 63°C annealing, and 30 s extension. Gel bands corresponding to target regions were purified using a QIAquick PCR Purification Kit (Qiagen, #28104), and purified DNA was cloned using a StrataClone Blunt PCR Cloning Kit (Stratagene, #240207) and plated on LB-Ampicillin agar with 40 µl 5-bromo-4-chloro-3-indolyl-β-D galactopyranoside (20 mg/ml). White colonies were cultured in 5 ml LB-Ampicillin broth and plasmids were purified with a QuickClean II Plasmid Miniprep Kit (GenScript, #L00420). Sequencing was performed at the Idaho State University Molecular Research Core Facility, using an Applied Biosystems 3130*xl* instrument and plasmid reactions purified with a BigDye XTerminator Purification Kit (Applied Biosystems, #4376485). Sequences were assembled and analyzed using Geneious Pro v5.5.6 software [54]. Intron regions were identified through comparative alignment of full-length genes with coding sequences. Promoter motif sequences, including transcription start sites, were identified using the Neural Network Promoter Prediction v2.2 software, Berkeley Drosophila Genome Project (http://www.fruitfly.org/seq_tools/promoter.html). Additional promoter motifs were identified using the PlantCARE database of cis-acting regulatory elements (http://bioinformatics.psb.ugent.be/webtools/plantcare/html)[55].

### Molecular marker development and application

Gene-specific, PCR-based markers were developed for *HGGT* and *VTE*4 based on sequence differences between Falcon and Azhul. Primers were designed to amplify a 50–80-bp region containing the polymorphism, and genotyping of the FA population was performed using high-resolution melt analysis, as described [56]. Markers were appended to the existing map using MultiPoint software (http://www.multiqtl.com) and QTL analysis was repeated separately for addition of each marker, using the same conditions as previously.

### Protein structure and gene expression

Secondary protein structure of *HGGT* was analyzed using coding sequences from Falcon and Azhul and the prediction function in Geneious Pro v5.4. Three-dimensional protein structure and function were analyzed using I-TASSER [57], [58].

Tissue for gene expression analysis was collected from etiolated shoots and mature embryos of Falcon and Azhul. Samples of each tissue type were germinated and maintained at two temperature treatments: room temperature and 4°C. RNA was extracted using an UltraClean Plant RNA Isolation Kit (Mo Bio, #13300-50). cDNA was synthesized using a SuperScript III First-Strand Synthesis System for RT-PCR (Invitrogen, #18080-051) primed with oligo(dT). Quantitative PCR was performed using 1x SSoFast EvaGreen Supermix (BioRad, #172-5201) with 80 ng cDNA and 0.5 µM forward and reverse primers in a 12.5-µl reaction volume. Thermocycling was performed on a BioRad C1000 thermal cycler with a CFX96 optics module, with initial denaturation at 98°C for 2 min followed by 40 cycles of 98°C for 2 s and 55°C for 5 s, with a fluorescence reading taken at the end of each cycle. Analysis of RT-PCR data was performed using a BioRad algorithm based the method of Vandesompele et al. [59].

## Supporting Information

Figure S1 **Identification of candidate genes influencing production of tocochromanols in the barley Falcon x Azhul mapping population.** Candidate genes were identified in rice chromosome regions syntenous with sequences of barley SNPs within the QTL.
(DOC)

Table S1 **Analysis of variance summaries for tocochromanol forms of Falcon, Azhul, Baronesse, and CDC Alamo.**
(DOC)

Table S2 **FASTA sequences.** Sequences for*VTE*4 and *HGGT* genes and promoter regions in Falcon, Azhul, and Falcon x Azhul RILs
(DOC)

Table S3 **Annotation of promoter regions.** A complete list of promoter motifs identified in the upstream regulatory regions of *VTE*4 and *HGGT*.
(XLS)

Table S4 **Falcon x Azhul SNPs coding an amino acid change in** ***HGGT*** **.**
(DOC)

Table S5 **Primer sequences used in cloning of gene and promoter regions, SNP genotyping, and quantitative PCR analysis in Falcon, Azhul, and the Falcon x Azhul RIL population.**
(DOC)

Table S6 **Genotype scores of** ***VTE*** **4 and** ***HGGT*** **markers in the Falcon x Azhul RIL population.** Genotyping was performed using high-resolution melt analysis of polymorphic regions within each gene.
(XLS)
