# Correction: Comparative Systems Biology Reveals Allelic Variation Modulating Tocochromanol Profiles in Barley (*Hordeum vulgare* L.)

**DOI:** 10.1371/journal.pone.0106020

**Published:** 2014-08-13

**Authors:** The PLOS ONE Staff

The email for corresponding author Eric W. Jackson is incorrect. The correct email is Eric.Jackson@genmills.com. The publisher apologizes for this error.
