# Supplementary figures and images for: Comparative Systems Biology Reveals Allelic Variation Modulating Tocochromanol Profiles in Barley (Hordeum vulgare L.)
Source: PLoS One. 2014 May 12;9(5):e96276. doi: 10.1371/journal.pone.0096276 (PMC4018352; doi:10.1371/journal.pone.0096276)

**Supplementary Figure 1**


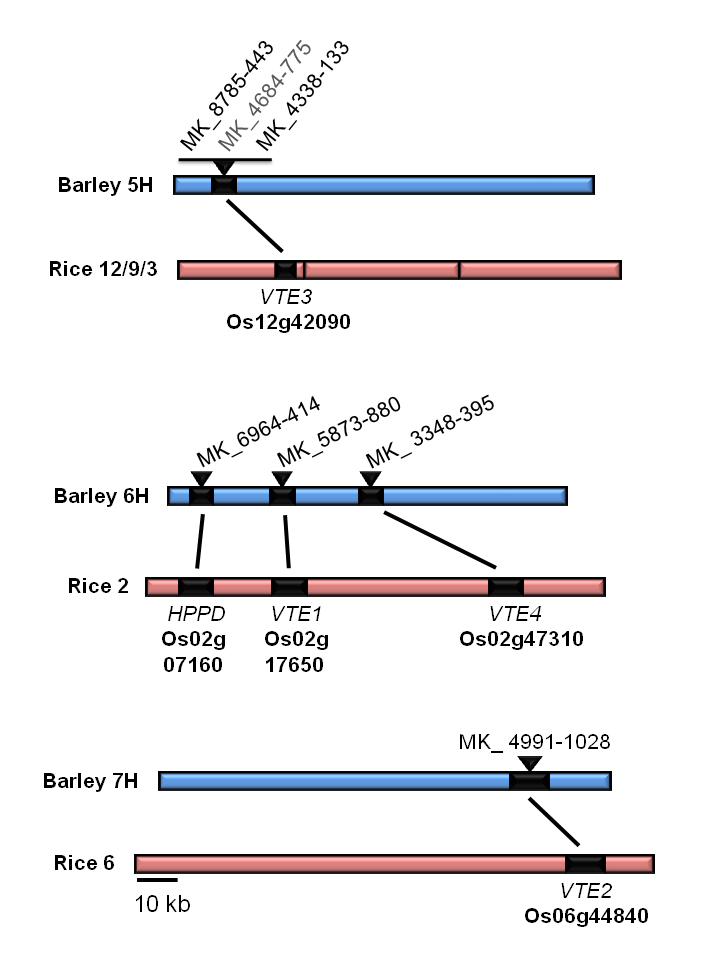

Supplement: Figure S1 — Identification of candidate genes influencing production of tocochromanols in the barley Falcon x Azhul mapping population. Candidate genes were identified in rice chromosome regions syntenous with sequences of barley SNPs within the QTL. (DOC) [file pone.0096276.s001.doc]
