# Supplementary material for: Comparative Systems Biology Reveals Allelic Variation Modulating Tocochromanol Profiles in Barley (Hordeum vulgare L.)
Source: PLoS One. 2014 May 12;9(5):e96276. doi: 10.1371/journal.pone.0096276 (PMC4018352; doi:10.1371/journal.pone.0096276)
Supplement: Table S1 — Analysis of variance summaries for tocochromanol forms of Falcon, Azhul, Baronesse, and CDC Alamo. (DOC) [file pone.0096276.s002.doc]

**Supplementary Table 1** Analysis of variance summaries for tocochromanol forms of Falcon, Azhul, Baronesse, and CDC Alamo

|  |  | | α | | | β | | | γ | | | δ | | | |  | |
| --- | --- | --- | --- | --- | --- | --- | --- | --- | --- | --- | --- | --- | --- | --- | --- | --- | --- |
| Tocochromanol | Source of variation | df | | SS | *P ^a^* | | SS | *P* | | SS | *P* | | SS | | *P* | | |
| Tocopherol | Genotype | 3 | | 26.38 | <0.0001* | | 0.83 | 0.0002* | | 253.87 | <0.0001* | | 6.08 | <0.0001* | | |  |
|  | Location | 1 | | 18.91 | <0.0001* | | 3.22 | <0.0001* | | 16.73 | 0.0001* | | 2.37 | <0.0001* | | |  |
|  | Genotype x Location | 3 | | 1.87 | 0.3950 | | 0.20 | 0.1750 | | 6.55 | 0.1113 | | 0.48 | 0.0087* | | |  |
|  | Year | 1 | | 100.85 | <0.0001* | | 0.02 | 0.5015 | | 75.10 | <0.0001* | | 3.71 | <0.0001* | | |  |
|  | Genotype x Year | 3 | | 2.45 | 0.2755 | | 0.23 | 0.1225 | | 47.68 | <0.0001* | | 1.02 | <0.0001* | | |  |
|  | Location x Year | 1 | | 5.77 | 0.0029* | | 0.43 | 0.0012* | | 14.94 | 0.0003* | | 0.34 | 0.0043* | | |  |
|  | Genotype x Loc. x Year | 3 | | 0.69 | 0.7765 | | 0.38 | 0.0240* | | 1.87 | 0.6273 | | 0.21 | 0.1626 | | |  |
| Tocotrienol | Genotype | 3 | | 1677.67 | <0.0001* | | 776.33 | <0.0001* | | 160.43 | <0.0001* | | 12.52 | <0.0001* | | |  |
|  | Location | 1 | | 635.19 | <0.0001* | | 56.75 | <0.0001* | | 3.24 | 0.1283 | | 1.13 | 0.0002* | | |  |
|  | Genotype x Location | 3 | | 64.53 | 0.0625 | | 29.65 | 0.0007* | | 10.29 | 0.0640 | | 2.18 | <0.0001* | | |  |
|  | Year | 1 | | 973.85 | <0.0001* | | 368.40 | <0.0001* | | 79.82 | <0.0001* | | 4.86 | <0.0001* | | |  |
|  | Genotype x Year | 3 | | 105.94 | 0.0081* | | 111.34 | <0.0001* | | 18.38 | 0.0054* | | 3.56 | <0.0001* | | |  |
|  | Location x Year | 1 | | 193.41 | <0.0001* | | 53.71 | <0.0001* | | 33.68 | <0.0001* | | 0.29 | 0.0500 | | |  |
|  | Genotype x Loc. x Year | 3 | | 43.71 | 0.1718 | | 20.00 | 0.0083* | | 2.01 | 0.6932 | | 0.36 | 0.1851 | | |  |

^a^ Asterisks indicate significance at p <0.05.
