# Supplementary material for: Comparative Systems Biology Reveals Allelic Variation Modulating Tocochromanol Profiles in Barley (Hordeum vulgare L.)
Source: PLoS One. 2014 May 12;9(5):e96276. doi: 10.1371/journal.pone.0096276 (PMC4018352; doi:10.1371/journal.pone.0096276)
Supplement: Table S2 — FASTA sequences. Sequences forVTE4 and HGGT genes and promoter regions in Falcon, Azhul, and Falcon x Azhul RILs (DOC) [file pone.0096276.s003.doc]

**Supplementary Table 2** Gene and promotor sequences for VTE4 and HGGT

>VTE4_gene_Falcon

ATGGAAAACTCCGCCGCCCTGCTCCACTCACTCCTCCTGTCCACGTCCTGGACGCCGCGCCGCCGTCTCGACCGAGCCTTGGCCACTCCCACGCGGCTCKCCCCGCCCGCCGGCCCGCTCTGCCGCTCCCGCCGGCCGACGCGATCTGTGCGCCCGATGGTGTCGTCGACGACCGCGGCCCGGGCCGACGCGGCGCCGCCGGGGCTGAAGGAAGGCATCGCGGGGCTCTACGACGAGTCGTCCGGCCTGTGGGAGAGCATCTGGGGCGAGCACATGCACCACGGCTTCTACGACTCTGGCGAGGCCGCCTCCATGTCCGACCACCGCCGCGCCCAGATCCGCATGATCGAGGAGGCCCTCGCTTTCGCCGCCGTCCCCGGTAAGTAACGTCCGGTCCAGACCCTATGCCCGCATCGAGATCGAACTATCTGATTGATTGAACCGTGTCGGCCCAATTCGTTGCTGGCCAGCCACTGCTGGGCTAAGGTATCCGACTGGCTGTACCTGCCACTTGCCCCGCTGGTGACCATTGCTTTTGCAAATACGAGATGGCACTGTTTGGATTGAGCTTGGTCGCCGTATCACTGTTCTGCTGGCTCTCTGCTCGCAGTCGTGGCACCAGCAGCAAGCCAATCTTGATTTGACCACAAAAATTTGCCTGAGCTGCGGATGTTTTTACAGTACGTTCTATCGCCCCAGTTAGCACTACTACCAGAGTGTGTCTTACAGTGGTAGAAGACCACACCGGTAATATACGTCTGAGAGTAGGCTGGTAGCCATTCCCTCCCCTATAAATTCTCTTCTGAACACTCCCTTTCAGTTCTGTAGAGTTTTTTTTTAGGTTTAATTCAAAATAGGTTTACGAGTTGCGACTTCTTTCTTTGAGAAATTAAGTGTGGGGTCTCCAGTAATTTTGGTGCTAGAGTGTGATTCCATTTTATGCCCACTCTCGACAGTCGACAATACATGCATGTTTTCTTTAATTGATATGGCATGAAAGGAATATCATAGATCTATCAGGTACTAGAGCTGCCTAAAAATCAATAACAGATATAAATATATGTTCAGTCATAAGTGTTACAGTTGCATAACCTCAACTACATCTTCAGTTCACTAGTATCTTGCAGCACAGTTCCATGGGAAATCAAGCTAGTTCAAAATAATAACAAAAGGTTAAAGGACACACTTAAAGAGAATGTAGCACCTTGTTCAAAACAAAGGGAACTTGGCCCATTGGTCGTTATATATGGACAATTAGCTTGATCGGAACTCCAAGTGCATAGTGCATGATTTATTGCTGTTATTGCTTGCGCTAATGCTTGTGCTTGCAGACGACCCGACAAACAAACCCAAAACAATTGTTGATGTTGGATGCGGAATCGGCGGTAGCTCAAGGTACCTGGCAAACAAATATGGAGCACAGTGCTCTGGGATCACATTGAGCCCAGTGCAAGCCGAGAGAGGAAATGCCCTAGCGGCGGCACAGGGGTTGGCAGACAAGGTTTTCTAATGCTTCACTCTTGGCAAATAGTTTGAGATTGCAATCTCAGACCTCATACGTACTTGGGGTATGGTTTTCCGTCGTTCGGTTTTACGTTGGAAGTGACAATGCCCACATGTTTGGCACAGGCTTCTTTCCAAGTTGCTGATGCTCTGGAGCAACCATTTCCTGATGGGCAGTTTGATCTTGTCTGGTCTATGGAGAGTGGTGAGCACATGCCGAACAAACAGAAGGTAAACATTCTAGTTCTAGAAGAAATTAAACATTGTGAGTCACTGCAAAACTGTTCACACGACAAGCTGTCAAAACATTATATCAGTTTGTGTGACAGACTGGGTCGATATATATCTCTTGCAAATAACTTAGTGTTGTTGGAAAGTCCGTTTTACCTATTTATGTAATTATCTTGTTTTCAAGAACATGTAATTACACTAATAAAGATACATAGTTAATTCCTATGACAAACGACAAACCCAGAAAATTATTCTGCTCAGGTATGAAAATAGGTGTTGAGTATTGTGTGAAATCTTAATAAATCTTTTATCAGTTTGTAGGTGAGCTGGCACGCGTCGCAGCTCCAGGAGCAACAATCATCATCGTGACCTGGTGCCATAGGAACCTTGCGCTATCTGAGGACTCACTGAAACCTGACGAGCTGAATCTTTTGAAAAAGATTTGTGATGCATATTACCTCCCGGATTGGTGCTCGCCCTCGGATTATGTCAAGATTGCCGAGTCATTGTCTCTTGAGGTATTTTCTCCCAAAATTTGAATTAATTTAACCTACCTGATACACAAATTTACATGATATCACAAAATATTCCTATACTGTCAGGATATCAAAACGGCTGACTGGTCTGAAAACGTGGCCCCGTTTTGGCCTGCTGTCATCCAATCAGCACTGACATGGAAAGGCCTCACTTCTCTACTAAGGAGTGGTACGTTCTTCTTAGCTCTCTCTCTATACTGTTCATCAAACCGAGATTATCTGATTCATGTTCTCTTTTTAGCCGATATACATTTTCAACTCACTTTGCTGTTGTCTTGATTATCAGGATGGAAGACGATAAAGGGAGCACTGGTGATGCCTCTCATGATCCAAGGCTACAAGAAAGGCCTGATCAAGTTCACCATCATCACCTGCCGCAAACCCCAAGCAGTCACAGAAGGAGAAGCTGAGACCGCATCGCCTAGTGTAGAATAG

>VTE4_gene_Azhul

ATGGAAAACTCCGCCGCCCTGCTCCACTCACTCCTCCTGTCCACGTCCTGGACGCCGCGCCGCCGTCTCGACCGAGCCTTGGCCACTCCCACGCGGCTCGCCCCGCCCGCCGGCCCGCTCTGCCGCTCCCGCCGGCCGACGCGATCTGTGCGCCCGATGGTGTCGTCGACGACCGCGGCCCGGGCCGACGCGGCGCCGCCGGGGCTGAAGGAAGGCATCGCGGGGCTCTACGACGAGTCGTCCGGCCTGTGGGAGAGCATCTGGGGCGAGCACATGCACCACGGCTTCTACGACTCTGGCGAGGCCGCCTCCATGTCCGACCACCGCCGCGCCCAGATCCGCATGATCGAGGAGGCCCTCGCTTTCGCCGCCGTCCCCGGTAAGTAACGTCCGGTCCAGACCCTATGCCCGCATCGAGATCGAACTATCTGATTGATTGAACCGTGTCGGCCCAATTCGTTGCTGGCCAGCCACTGCTGGGCTAAGGTATCCGACTGGCTGTACCTGCCACTTGCCCCGCTGGTGACCATTGCTTTTGCAAATACGAGATGGCACTGTTTGGATTGAGCTTGGTCGCCGTATCACTGTTCTGCTGGCTCTCTGCTCGCAGTCGTGGCACCAGCAGCAAGCCAATCTTGATTTGACCACAAAAATTTGCCTGAGCTGCGGATGTTTTTACAGTACGTTCTATCGCCCCAGTTAGCACTACTACCAGAGTGTGTCTTACAGTGGTAGAAGACCACACCGGTAATATACGTCTGAGAGTAGGCTGGTAGCCATTCCCTCCCCTATAAATTCTCTTCTGAACACTCCCTTTCAGTTCTGTAGAGTTTTTTTTTAGGTTTAATTCAAAATAGGTTTACGAGTTGCGACTTCTTTCTTTGAGAAATTAAGTGTGGGGTCTCCAGTAATTTTGGTGCTAGAGTGTGATTCCATTTTATGCCCACTCTCGACAGTCGACAATACATGCATGTTTTCTTTAATTGATATGGCATGAAAGGAATATCATAGATCTATCAGGTACTAGAGCTGCCTAAAAATCAATAACAGATATAAATATATGTTCAGTCATAAGTGTTACAGTTGCATAACCTCAACTACATCTTCAGTTCACTAGTATCTTGCAGCACAGTTCCATGGGAAATCAAGCTAGTTCAAAATAATAACAAAAGGTTAAAGGACACACTTAAAGAGAATGTAGCACCTTGTTCAAAACAAAGGGAACTTGGCCCATTGGTCGTTATATATGGACAATTAGCTTGATCGGAACTCCAAGTGCATAGTGCATGATTTATTGCTGTTATTGCTTGCGCTAATGCTTGTGCTTGCAGACGACCCGACAAACAAACCCAAAACAATTGTTGATGTTGGATGCGGAATCGGCGGTAGCTCAAGGTACCTGGCAAACAAATATGGAGCACAGTGCTCTGGGATCACATTGAGCCCAGTGCAAGCCGAGAGAGGAAATGCCCTAGCGGCGGCACAGGGGTTGGCAGACAAGGTTTTCTAATGCTTCACTCTTGGCAAATAGTTTGAGATTGCAATCTCAGACCTCATACGTACTTGGGGTATGGTTTTCCGTCGTTCGGTTTTACGTTGGAAGTGACAATGCCCACATGTTTGGCACAGGCTTCTTTCCAAGTTGCTGATGCTCTGGAGCAACCATTTCCTGATGGGCAGTTTGATCTTGTCTGGTCTATGGAGAGTGGTGAGCACATGCCGAACAAACAGAAGGTAAACATTCTAGTTCTAGAAGAAATTAAACATTGTGAGTCACTGCAAAACTGTTCACGACAAGCTGTCAAAACATTATATCAGTTTGTGTGACAGACTGGGTCGATATATATCTCTTGCAAATAACTTAGTGTTGTTGGAAAGTCCGTTTTACCTATTTATGTAATTATCTTGTTTTCAAGAACATGTAATTACACTAATAAAGATACATAGTTAATTCCTATGACAAACGACAAACCCAGAAAATTATTCTGCTCAGGTATGAAAATAGGTGTTGAGTATTGTGTGAAATCTTAATAAATCTTTTATCAGTTTGTAGGTGAGCTGGCACGCGTCGCAGCTCCAGGAGCAACAATCATCATCGTGACCTGGTGCCATAGGAACCTTGCGCTATCTGAGGACTCACTGAAACCTGACGAGCTGAATCTTTTGAAAAAGATTTGTGATGCATATTACCTCCCGGATTGGTGCTCGCCCTCGGATTATGTCAAGATTGCCGAGTCATTGTCTCTTGAGGTATTTTCTCCCAAAATTTGAATTAATTTAACCTACCTGATACACAAATTTACATGATATCACAAAATATTCCTATACTGTCAGGATATCAAAACGGCTGACTGGTCTGAAAACGTGGCCCCGTTTTGGCCTGCTGTCATCCAATCAGCACTGACATGGAAAGGCCTCACTTCTCTACTAAGGAGTGGTACGTTCTTCTTAGCTCTCTCTCTATACTGTTCATCAAACCGAGATTATCTGATTCATGTTCTCTTTTTAGCCGATATACATTTTCAACTCACTTTGCTGTTGTCTTGATTATCAGGATGGAAGACGATAAAGGGAGCACTGGTGATGCCTCTCATGATCCAAGGCTACAAGAAAGGCCTGATCAAGTTCACCATCATCACCTGCCGCAAACCCCAAGCAGTCACAGAAGGAGAAGCTGAGACCGCATCGCCTAGTGTAGAATAG

>VTE4_promoter_region_Falcon

CATATATTGTATACGCATCAGAAAGAATATAATAGGAAATCTAGAGCACATCACGCAGTCAATTGTGTTACACACCGCACGCCCCTTCGTGGGCCCATATTTGTTTTGGTTTGATGCTAAGATGGATAGGCTATCGACGGTTGGTTGTCTGCCGTTATTCTATTTCTAGCAATTTTGTCCATGCATCGTCTCTCTAAGTCTCCATCCCTCTCTTTCCACGCAAAGTCCCTTCCTACCCTAGATCTGTGTCGCTTGGAGGCACTCGAGGTAACTGCGCAAGTGCGGCGGCCGGCGACCGGCAACCAGCGTAGCCAAGGTTGCGAGGCAAGCTCCTGCCTCTCCAGGAGCTCGATTTCGACGAGACTCACAGGCGAGGGAGCGGAGGAGATCTCAATTCTCCGTGCGCGAGCTCGTTGGCGTGGAGTCGCACCAATGATCTTTTTTTTAAGATTTCAGGACTGCCTGACCTATAGCCGATGGGGAGGAAGATGAGCGTGGCTGAACGCACAGTTGCCTGGGGAGCGACAGGCGATTTCGGTAGCGAGGAGTTTCGAGGCTTGATATATTGACTGGTGTTAATTTATATAGTCATGGATCGGGCCGTGATGCTTCGAGGCTCGTGCAGAGTCCTTGTCCGAAACTGGTCAAACGAGGCGAGATCATGCGGAGAACGACACTCGTTTTTAATTATTATTAGTGCGCTTGGGTCCGGTGCGCTTGTGTCATTCATGAACAAAACGGTTTAAACTGCGGGTTGAATAACAAAAATTACAGGGGCTTTTTTATAAAAATGTTGTGGTGAATTTTCAATAGAAGCAATAGCCTTTTTATTATTAAAAAAATAAAGTATAGATGTATATTAGCCTGTATATCTTGAAAACGCTTTATAAAAAGGAATCTCGAAGTCCTTTCCCACGAAGGGGATACCGAGGACGCTTGGCCTCTGCAACACCGTTCTCAGGCTAACCGTGCCCTCTTCAGCCCCGCCGGTATATGCTGAGCTGCCATTACGTGGCCCACGTGACCCCGCGTCGTTAAAAAAGTACTGTATTGCCTCGGGAGCTGTCAGTCAGGCGCTCACGGGGTCTCCAAATACAAAATG

>VTE4_promoter_region_Azhul

CATATATTGTATACGCATCAGAAAGAATATAATAGGAAATCTAGAGCACATCACGCAGTCAATTGTGTTACACACCGCACGCCCCTTCGTGGGCCCATATTTGTTTTGGTTTGATGCTAAGATGGATAGGCTATCGACGGTTGGTTGTCTGCCGTTATTCTATTTCTAGCAATTTTGTCCATGCATCGTCTCTCTAAGTCTCCATCCCTCTCTTTCCACGCAAAGTCCCTTCCTACCCTAGATCTGTGTCGCTTGGAGGCACTCGAGGTAACTGCGCAAGTGCGGCGGCCGGCGACCGGCAACCAGCGTAGCCAAGGTTGCGAGGCAAGCTCCTGCCTCTCCAGGAGCTCGATTTCGACGAGACTCACAGGCGAGGGAGCGGAGGAGATCTCAATTCTCCGTGCGCGAGCTCGTTGGCGTGGAGTCGCACCAATGATCTTTTTTTTAAGATTTCAGGACTGCCTGACCTATAGCCGATGGGGAGGAAGATGAGCGTGGCTGAACGCACAGTTGCCTGGGGAGCGACAGGCGATTTCGGTAGCGAGGAGTTTCGAGGCTTGATATATTGACTGGTGTTAATTTATATAGTCATGGATCGGGCCGTGATGCTTCGAGGCTCGTGCAGAGTCCTTGTCCGAAACTGGTCAAACGAGGCGAGATCATGCGGAGAACGACACTCGTTTTTAATTATTATTAGTGCGCTTGGGTCCGGTGCGCTTGTGTCATTCATGAACAAAACGGTTTAAACTGCGGGTTGAATAACAAAAATTATAGGGGCTTTTTTATAAAAATGTTGTGGTGAATTTCCAATAGAAGCAATAGCCTTTTTATTATTAAAAAAATAAAGTATAGATGTATATTAGCCTGTATATCTTGAAAACGCTTTATAAAAAGGAATCTCGAAGTCCTTTCCCACGAAGGGGATACCGAGGACGCTTGGCCTCTGCAACACCGTTCTCAGGCTAACCGTGCCCTCTTCAGCCCCGCCGGTATATGCTGAGCTGCCATTACGTGGCCCACGTGACCCCGCGTCGTTAAAAAAGTACTGTATTGCCTCGGGAGCTGTCAGTCAGGCGCTCACGGGGTCTCCAAATACAAAATG

>HGGT_gene_Falcon

ATGCAAGCCGTCACGGCGGCGGCCGCGGCGGGGCAGCTGCTCACAGGCAATCACGTCTCCTCGATCGACATATTAGTCTTCCTTGCCTCGGAGAGATCTCGCTCGGGGTCTTTTTGTTTGGGTTAGAGATTAGATTAGGTTTTGTTTTTCGAACTGAGGGAGGGTTATATATATTTGATTTGATTTGATTTGATTTGATCTAGTTTGGTTCGGATTGAGGGTTCTTATCTTGACTCGGCATCCGCCGTTTGGTCTTGTGTATGTGATGCAGATACGAGGAGAGGGCCCAGATGTAGGGCTCGGCTGGGAACGACGAGATTGTCCTGGACAGGTATACGTCTGTTCTGTATCTTGAATTCTTGATTGATGATTTGCCTGCTGGTCCTCTTGTTTTTATCTTCTTTGGGTACAAAAGATCAACATTTCCAGTCAGGAAAAATGAGAACTGGAAAATAAGGGGGACAAATCGAACCTCTGAACCGCAGATATGGATTCAGGGATTGCATAATTGACAACATTGTGTGCTGTTATGTTTTCTTCTACTGAGGGATATATATGTTGCTATTTAGAATAGAGGATGGGATTCAAAATGAGTGCCAGGAAAACAAAATTTCTCTCTATATATATGAAGGAACTGGAATTTTTTTTCTTTTAGAAAAGGAGGCGACGCCGCCGCCTTTGCATCGAGGGATGCATGCGGCCATGGAGGAACTGGAATTATTTTTCTTTTAGAAAAGGAGGCGACGCCGCGGCCTTTGCATCGAGGGATACATGCGGCCATGGAGGAACTGGAATTTTTTTTCTTTTAGAAAAGGAGGCGACGCCGCGGCCTTTGCATCGAGGGATGCATGCGGCCATGGAGGAACTGGAATATGCCATGTCTTCGGTTGGTGTTTCTTTTACAACAGATTAACAGCATAAAAGAGGTTTTCTGTTGATAGAAGTGTTGGATATCCGTAATTTCTTACCCAAATGGAAGTTATCAAGTCTGTTTTTTTCCTCCCCAATTCAATAACCGTTGTGTCGTCCATTTTGCAAATTACAGGTCGATTTGCAGTGGAAGCTTTTGCAGGCCAGTGCCAAAGGTAAGTAAATCGTCTTCCGATCCATGTGACGTCTCATTTATCGCCATGCATCTTGCTAATCTCCGTTGGCTTCCTTCTAAACAGCAGCGCTACTGCTGTAATGCATAAATTCAGTGCCATTTCGCAAGCTGCTATGCCTAGAAGAAACACAAAGAGGCAGTGCAGCGATGATTATCCAGCCCTCCAAGCTGGATGCAGCGAGGTTAATTGGGATCGAAACGGCTCCAACGCCAATCGGCTTGAGGAAATCAGGGGAGATGTTTTGAAGAAATTGCGCTCTTTCTATGAATTTTGCAGGCCACACACAATTTTTGGCACTGTGAGCTCTGGCATCTTTGATCCAAATTTACACATGTATAGTTCTGATGCACACTGTCCAATGCAATGTTTTTTATGTGGACTATATTGTTAGATAATAGGTATAACTTCAGTGTCTCTCCTGCCAATGAAGAGCATAGATGATTTTACTGTCACGGTACTACGAGGATATCTCGAGGTATTATAAATTTGCATCTCCTCTGAGTACTATGCTTTAGCAATATACATAACCATTTGTTATATGATAAATTTACACCGTCAACTCTTATAAAGAGCTCTAAAATTTCCTCTTTGTGTGCAGGCTTTGACTGCTGCTTTATGTATGAACATTTATGTGGTCGGGCTGAATCAGCTATATGACATTCAGATTGACAAGGTAGTATTTTTTCCTTGTGATTTCAAAAATTAAATGTACCTTCTTAATCCTTTATATCGTAGAATCATTTATACTAAATTATACCCTGTTTTGGAGATCAACAAGCCAGGTCTTCCATTGGCATCTGGGGAATTTTCAGTAGCAACTGGAGTTTTCTTAGTACTCGCATTCCTGATCATGGTATATTAGGTTCAACACAACATTGTTCTAGTGTGAGAAAAATAGGCTTCATGTCTAAATACCTTAAGTTTCGTTGTTTTCAGAGCTTTAGCATAGGAATACGTTCCGGATCGGCGCCACTGATGTGTGCTTTAATTGTCAGCTTCCTTCTTGGAAGTGCGTACTCCATTGAGGTAAGTAGCTTTTACCATGATGTAAATTGCAAAGTAAGTTCCATGAATCCATCAAATGTCAAGTGGTGATCATCTTCCTCATATTTTATCTTTTTCAGGCTCCGTTCCTCCGGTGGAAACGGCACGCGCTCCTCGCTGCATCATGTATCCTATTTGTGAGGGCTATCTTGGTCCAGTTGGCTTTCTTTGCACATATGCAGGTACTTAGCACAGTACATATCTTCAGTAGCACTGGAAATTCATACTTATTTGGCTTCCTTCATTGTTGTTTGATGATTCATTTGGCAGCAACATGTTCTGAAAAGGCCATTGGCAGCAACCAAATCGCTGGTGTTTGCAACATTGTTTATGTGTTGCTTCTCTGCCGTCATAGCACTATTCAAGGTAATTAAACATGCAATGACTTAATAGTTTTGGCATCATCATACGTATCTTTTAATTTATGTCCTGAACTTTCAGGATATTCCAGATGTTGATGGAGACCGAGACTTTGGTATCCAATCCTTGAGTGTGAGATTGGGGCCTCAAAGAGTAAGAGCAGACCCGCATATCTCAGGAAGTGTTTCGAGTAAAACATATGCCTCTGTAAATTGGTACCCCTTTTGAACTTATTTTTTCCTACTGTAGGTGTATCAGCTCTGCATAAGCATATTGTTGACAGCCTATGGCGCTGCCACTCTAGTAGGAGCTTCATCCACAAACCTATTTCAAAAGATCATCACTGTGAGTTATCCGTTGTAAAATCCAGAATTTGAATGAAATACAAACAATTTATAAGCTAAGAAATCTTCCATTGTTGCAATTTTGCATGCTTTGATCATCCAGGTGTCTGGTCATGGCCTGCTTGCTTTGACACTTTGGCAGAGAGCGCAGCACTTTGAGGTTGAAAACCAAGCGCGTGTCACATCATTTTACATGTTCATTTGGAAGGTAATTAATTAAGTTGCTGGCTTATATTGTGCATTCTCTGGACCATTAGACTTTGACTATGTATGCCTAATGATTATTTGCACGTTTTGTGTCACCTTCATGCAGCTATTCTATGCAGAGTATTTCCTTATACCATTTGTGCAGTGA

>HGGT_gene_Azhul

ATGCAAGCCGTCACGGCGGCGGCCGCGGCGGGGCAGCTGCTAACAGGCAATCACGTCTCCTCGATCGACATATTAGTCTTCCTTGCCTCGGAGAGATCTCGCTCGGGTCTTTTTGTTTGGGTTAGAGATTAGATTAGGTTTTGTTTTTCGAACTGAGGGAGGGTTATATAGATTTGATTTGATGAGATTTGATTTGATCTGGTTTGGTTCGGATTGAGGGTTCTTATCTTGACTCGGCATCCGCCGTTCCGTCTTGTGTATGTGATGCAGATACGAGGAGAGGGCCCAGATGTAGGGCTCGGCTGGGAACGACGAGATTATCCTGGACAGGTATACGTCCGTTCTGTATCTTGAATTCTTGATTGATGATTTGCCTGCTGGTGCTGTTGTTTTTATCTTCTTTGGGTACAAATCAACATTTCCAGTCAGGAAAAATGAGAACTGGAAAATAAGGGGGACAAATCGAACCTCCGAACCGCAGATATGGATTCAGAGATCGCATGATTGACAACATTGTGTGCTGTTATGTTTTATTCTACTGAGGGATATATATGTTGCTATTTAGAATAGAGGATGGGATTCAAAATGAGTGCCAGGAAAATAAATTTCTCTATATATATATGGAGGAACTGGAATTTTCTTTTTTTAGAAAAGGAGGCGACGCCCCGGCCTCTGCATCGAGGGATGCATGCGGCCATGGAGGAACTGGAATATGCCATGTCTTCGGTTGGTGTTTCTTTTACAACAGATTAACAGCATAAAAGAGGTTTTCTGTTGATAGAAGTGTTGGAATATCCGTAGTTTCTTACCCAAATGGAAGTTATCAAGTCTGTTTTTTTCCTCCCCAATTCAATAACCGTTGTGTCGTCCATTTTGCAAATTACAGGTCGATTTGCAGTGGAAGCTTTTGCAGGCCAGTGCCAAAGGTAAGTAAATCGTCTTCCGATCCATGTGACGTCTTGTTTATCGCCATGCATCTTGCTAATCTCCGTTGGCTTCCTTCTAAACAGCAGTGCTACTACTGTAATGCATAAATTCAGTGCCATTTCTCAAGCTGCTAGGCCTAGAAGAAACACAAAGAGACAGTGCAGCGATGATTATCCAGCCCTCCAAGCTGGATGCAGCGAGGTTAATTGGGATCAAAACGGTTCCAACGCCAATCGGCTTGAGGAAATCAGGGGAGATGTTTTGAAGAAATTGCGCTCTTTCTATGAATTTTGCAGGCCACACACAATTTTTGGCACTGTGAGCTCTGGCATCTTTGATCCAAATTTACACATGTATAGTTCTGATGCACACTGTCCAATGCAATGTTTCTTATGTGGACTATATTGTTAGATAATAGGTATAACTTCAGTGTCTCTCCTGCCAATGAAGAGCATAGATGATTTTACTGTCACGGTACTACGAGGATATCTCGAGGTATTATAAATTTGCATCTCCTCTGAGTACTATGCTTTAGCAATATACATAACCATTTGTTATATGATAAATTTACACCGTCAACTCTTATAAAGAGCTCTAAAATTTCCTCTTTGTGTGCAGGCTTTGACTGCTGCTTTATGTATGAACATTTATGTGGTCGGGCTGAATCAGCTATATGACATTCAGATTGACAAGGTAGTATTTTTTCCTTGTGATTTCAAAAATTAAATGTACCTTCTTAATCCTTTATATCGTAGAATCATTTATACTAAATTATACCCTGTTTTGGAGATCAACAAGCCAGGTCTTCCATTGGCATCTGGGGAATTTTCAGTAGCAACTGGAGTTTTCTTAGTACTCGCATTCCTGATCATGGTATATTAGGTTCAACACAACATTGTTCTAGTGTGAGAAAAATAGGCTTCATGTCTAAATACCTTAAGTTTCGTTGTTTTCAGAGCTTTAGCATAGGAATACGTTCCGGATCGGCGCCACTGATGTGTGCTTTAATTGTCAGCTTCCTTCTTGGAAGTGCGTACTCCATTGAGGTAAGTAGCTTTTACCATGATGTAAATTGCAAAGTAAGTTCCATGAATCCATCAAATGTCAAGTGGTGATCATCTTCCTCATATTTTATCTTTTTCAGGCTCCGTTCCTCCGGTGGAAACGGCACGCGCTCCTCGCTGCATCATGTATCCTATTTGTGAGGGCTATCTTGGTCCAGTTGGCTTTCTTTGCACATATGCAGGTACTTAGCACAGTACATATCTTCAGTAGCACTGGAAATTCATACTCATTTGGCTTCCTTCATTGTTGTTTGATGATTCATTTGGCAGCAACATGTTCTGAAAAGGCCATTGGCAGCAACCAAATCGCTGGTGTTTGCAACATTGTTTATGTGTTGCTTCTCTGCCGTCATAGCACTATTCAAGGTAATTAAACATGCAATGACTTAATAGTTTTGGCATCATCATACGTATCTTTTAATTTATGTCCTGAACTTTCAGGATATTCCAGATGTTGATGGAGATCGAGACTTTGGTATCCAATCCTTGAGTGTGAGATTGGGGCCTCAAAGAGTAAGAACAGACCCGCATATCTCAGGAAGTGTTTCGAGTAAAACATATGCCTCTGTAAATTGGTACCCCTTTTGAACTTATTTTTTCCTACTGTAGGTGTATCAGCTCTGCATAAGCATATTGTTGACAGCCTATGGCGCTGCCACTCTAGTAGGAGCTTCATCCACAAACCTATTTCAAAAGATCATCACTGTGAGTTATCCGTTGTAAAATCCAGAATTTGAATGAAATACAAACAATTTATAAGCTAAGAAATCTTCCATTGTTGCAATTTTGCATGCTTTGATCATCCAGGTGTCTGGTCATGGCCTGCTTGCTTTGACACTTTGGCAGAGAGCGCAGCACTTTGAGGTTGAAAACCAAGCGCGTGTCACATCATTTTACATGTTCATTTGGAAGGTAATTAATTAAGTTGCTGGCTTATATTGTGCATTCTCTGGACCATTAGACTTTGACTATGTATGCCTAATGATTATTTGCACGTTTTGTGTCACCTTCATGCAGCTATTCTATGCAGAGTATTTCCTTATACCATTTGTGCAGTGA

>HGGT_gene_FA.41

ATGCAAGCCGTCACGGCGGCGGCCGCGGCGGGGCAGCTGCTCACAGGCAATCACGTCTCCTCGATCGACATATTAGTCTTCCTTGCCTCGGAGAGATCTCGCTCGGGGTCTTTTTGTTTGGGTTAGAGATTAGATTAGGTTTTGTTTTTCGAACTGAGGGAGGGTTATATATATTTGATTTGATTTGATTTGATTTGATCTAGTTTGGTTCGGATTGAGGGTTCTTATCTTGACTCGGCATCCGCCGTTTGGTCTTGTGTATGTGATGCAGATACGAGGAGAGGGCCCAGATGTAGGGCTCGGCTGGGAACGACGAGATTGTCCTGGACAGGTATACGTCTGTTCTGTATCTTGAATTCTTGATTGATGATTTGCCTGCTGGTCCTCTTGTTTTTATCTTCTTTGGGTACAAAAGATCAACATTTCCAGTCAGGAAAAATGAGAACTGGAAAATAAGGGGGACAAATCGAACCTCTGAACCGCAGATATGGATTCAGGGATTGCATAATTGACAACATTGTGTGCTGTTATGTTTTCTTCTACTGAGGGATATATATGTTGCTATTTAGAATAGAGGATGGGATTCAAAATGAGTGCCAGGAAAACAAAATTTCTCTCTATATATATGAAGGAACTGGAATTTTTTTTCTTTTAGAAAAGGAGGCGACGCCGCCGCCTTTGCATCGAGGGATGCATGCGGCCATGGAGGAACTGGAATTATTTTTCTTTTAGAAAAGGAGGCGACGCCGCGGCCTTTGCATCGAGGGATACATGCGGCCATGGAGGAACTGGAATTTTTTTTCTTTTAGAAAAGGAGGCGACGCCGCGGCCTTTGCATCGAGGGATGCATGCGGCCATGGAGGAACTGGAATATGCCATGTCTTCGGTTGGTGTTTCTTTTACAACAGATTAACAGCATAAAAGAGGTTTTCTGTTGATAGAAGTGTTGGATATCCGTAATTTCTTACCCAAATGGAAGTTATCAAGTCTGTTTTTTTCCTCCCCAATTCAATAACCGTTGTGTCGTCCATTTTGCAAATTACAGGTCGATTTGCAGTGGAAGCTTTTGCAGGCCAGTGCCAAAGGTAAGTAAATCGTCTTCCGATCCATGTGACGTCTCATTTATCGCCATGCATCTTGCTAATCTCCGTTGGCTTCCTTCTAAACAGCAGCGCTACTGCTGTAATGCATAAATTCAGTGCCATTTCGCAAGCTGCTATGCCTAGAAGAAACACAAAGAGGCAGTGCAGCGATGATTATCCAGCCCTCCAAGCTGGATGCAGCGAGGTTAATTGGGATCGAAACGGCTCCAACGCCAATCGGCTTGAGGAAATCAGGGGAGATGTTTTGAAGAAATTGCGCTCTTTCTATGAATTTTGCAGGCCACACACAATTTTTGGCACTGTGAGCTCTGGCATCTTTGATCCAAATTTACACATGTATAGTTCTGATGCACACTGTCCAATGCAATGTTTTTTATGTGGACTATATTGTTAGATAATAGGTATAACTTCAGTGTCTCTCCTGCCAATGAAGAGCATAGATGATTTTACTGTCACGGTACTACGAGGATATCTCGAGGTATTATAAATTTGCATCTCCTCTGAGTACTATGCTTTAGCAATATACATAACCATTTGTTATATGATAAATTTACACCGTCAACTCTTATAAAGAGCTCTAAAATTTCCTCTTTGTGTGCAGGCTTTGACTGCTGCTTTATGTATGAACATTTATGTGGTCGGGCTGAATCAGCTATATGACATTCAGATTGACAAGGTAGTATTTTTTCCTTGTGATTTCAAAAATTAAATGTACCTTCTTAATCCTTTATATCGTAGAATCATTTATACTAAATTATACCCTGTTTTGGAGATCAACAAGCCAGGTCTTCCATTGGCATCTGGGGAATTTTCAGTAGCAACTGGAGTTTTCTTAGTACTCGCATTCCTGATCATGGTATATTAGGTTCAACACAACATTGTTCTAGTGTGAGAAAAATAGGCTTCATGTCTAAATACCTTAAGTTTCGTTGTTTTCAGAGCTTTAGCATAGGAATACGTTCCGGATCGGCGCCACTGATGTGTGCTTTAATTGTCAGCTTCCTTCTTGGAAGTGCGTACTCCATTGAGGTAAGTAGCTTTTACCATGATGTAAATTGCAAAGTAAGTTCCATGAATCCATCAAATGTCAAGTGGTGATCATCTTCCTCATATTTTATCTTTTTCAGGCTCCGTTCCTCCGGTGGAAACGGCACGCGCTCCTCGCTGCATCATGTATCCTATTTGTGAGGGCTATCTTGGTCCAGTTGGCTTTCTTTGCACATATGCAGGTACTTAGCACAGTACATATCTTCAGTAGCACTGGAAATTCATACTTATTTGGCTTCCTTCATTGTTGTTTGATGATTCATTTGGCAGCAACATGTTCTGAAAAGGCCATTGGCAGCAACCAAATCGCTGGTGTTTGCAACATTGTTTATGTGTTGCTTCTCTGCCGTCATAGCACTATTCAAGGTAATTAAACATGCAATGACTTAATAGTTTTGGCATCATCATACGTATCTTTTAATTTATGTCCTGAACTTTCAGGATATTCCAGATGTTGATGGAGACCGAGACTTTGGTATCCAATCCTTGAGTGTGAGATTGGGGCCTCAAAGAGTAAGAGCAGACCCGCATATCTCAGGAAGTGTTTCGAGTAAAACATATGCCTCTGTAAATTGGTACCCCTTTTGAACTTATTTTTTCCTACTGTAGGTGTATCAGCTCTGCATAAGCATATTGTTGACAGCCTATGGCGCTGCCACTCTAGTAGGAGCTTCATCCACAAACCTATTTCAAAAGATCATCACTGTGAGTTATCCGTTGTAAAATCCAGAATTTGAATGAAATACAAACAATTTATAAGCTAAGAAATCTTCCATTGTTGCAATTTTGCATGCTTTGATCATCCAGGTGTCTGGTCATGGCCTGCTTGCTTTGACACTTTGGCAGAGAGCGCAGCACTTTGAGGTTGAAAACCAAGCGCGTGTCACATCATTTTACATGTTCATTTGGAAGGTAATTAATTAAGTTGCTGGCTTATATTGTGCATTCTCTGGACCATTAGACTTTGACTATGTATGCCTAATGATTATTTGCACGTTTTGTGTCACCTTCATGCAGCTATTCTATGCAGAGTATTTCCTTATACCATTTGTGCAGTGA

>HGGT_gene_FA.117

ATGCAAGCCGTCACGGCGGCGGCCGCGGCGGGGCAGCTGCTAACAGGCAATCACGTCTCCTCGATCGACATATTAGTCTTCCTTGCCTCGGAGAGATCTCGCTCGGGTCTTTTTGTTTGGGTTAGAGATTAGATTAGGTTTTGTTTTTCGAACTGAGGGAGGGTTATATAGATTTGATTTGATGAGATTTGATTTGATCTGGTTTGGTTCGGATTGAGGGTTCTTATCTTGACTCGGCATCCGCCGTTCCGTCTTGTGTATGTGATGCAGATACGAGGAGAGGGCCCAGATGTAGGGCTCGGCTGGGAACGACGAGATTATCCTGGACAGGTATACGTCCGTTCTGTATCTTGAATTCTTGATTGATGATTTGCCTGCTGGTGCTGTTGTTTTTATCTTCTTTGGGTACAAATCAACATTTCCAGTCAGGAAAAATGAGAACTGGAAAATAAGGGGGACAAATCGAACCTCCGAACCGCAGATATGGATTCAGAGATCGCATGATTGACAACATTGTGTGCTGTTATGTTTTATTCTACTGAGGGATATATATGTTGCTATTTAGAATAGAGGATGGGATTCAAAATGAGTGCCAGGAAAATAAATTTCTCTATATATATATGGAGGAACTGGAATTTTCTTTTTTTAGAAAAGGAGGCGACGCCCCGGCCTCTGCATCGAGGGATGCATGCGGCCATGGAGGAACTGGAATATGCCATGTCTTCGGTTGGTGTTTCTTTTACAACAGATTAACAGCATAAAAGAGGTTTTCTGTTGATAGAAGTGTTGGAATATCCGTAGTTTCTTACCCAAATGGAAGTTATCAAGTCTGTTTTTTTCCTCCCCAATTCAATAACCGTTGTGTCGTCCATTTTGCAAATTACAGGTCGATTTGCAGTGGAAGCTTTTGCAGGCCAGTGCCAAAGGTAAGTAAATCGTCTTCCGATCCATGTGACGTCTTGTTTATCGCCATGCATCTTGCTAATCTCCGTTGGCTTCCTTCTAAACAGCAGTGCTACTACTGTAATGCATAAATTCAGTGCCATTTCTCAAGCTGCTAGGCCTAGAAGAAACACAAAGAGACAGTGCAGCGATGATTATCCAGCCCTCCAAGCTGGATGCAGCGAGGTTAATTGGGATCAAAACGGTTCCAACGCCAATCGGCTTGAGGAAATCAGGGGAGATGTTTTGAAGAAATTGCGCTCTTTCTATGAATTTTGCAGGCCACACACAATTTTTGGCACTGTGAGCTCTGGCATCTTTGATCCAAATTTACACATGTATAGTTCTGATGCACACTGTCCAATGCAATGTTTCTTATGTGGACTATATTGTTAGATAATAGGTATAACTTCAGTGTCTCTCCTGCCAATGAAGAGCATAGATGATTTTACTGTCACGGTACTACGAGGATATCTCGAGGTATTATAAATTTGCATCTCCTCTGAGTACTATGCTTTAGCAATATACATAACCATTTGTTATATGATAAATTTACACCGTCAACTCTTATAAAGAGCTCTAAAATTTCCTCTTTGTGTGCAGGCTTTGACTGCTGCTTTATGTATGAACATTTATGTGGTCGGGCTGAATCAGCTATATGACATTCAGATTGACAAGGTAGTATTTTTTCCTTGTGATTTCAAAAATTAAATGTACCTTCTTAATCCTTTATATCGTAGAATCATTTATACTAAATTATACCCTGTTTTGGAGATCAACAAGCCAGGTCTTCCATTGGCATCTGGGGAATTTTCAGTAGCAACTGGAGTTTTCTTAGTACTCGCATTCCTGATCATGGTATATTAGGTTCAACACAACATTGTTCTAGTGTGAGAAAAATAGGCTTCATGTCTAAATACCTTAAGTTTCGTTGTTTTCAGAGCTTTAGCATAGGAATACGTTCCGGATCGGCGCCACTGATGTGTGCTTTAATTGTCAGCTTCCTTCTTGGAAGTGCGTACTCCATTGAGGTAAGTAGCTTTTACCATGATGTAAATTGCAAAGTAAGTTCCATGAATCCATCAAATGTCAAGTGGTGATCATCTTCCTCATATTTTATCTTTTTCAGGCTCCGTTCCTCCGGTGGAAACGGCACGCGCTCCTCGCTGCATCATGTATCCTATTTGTGAGGGCTATCTTGGTCCAGTTGGCTTTCTTTGCACATATGCAGGTACTTAGCACAGTACATATCTTCAGTAGCACTGGAAATTCATACTCATTTGGCTTCCTTCATTGTTGTTTGATGATTCATTTGGCAGCAACATGTTCTGAAAAGGCCATTGGCAGCAACCAAATCGCTGGTGTTTGCAACATTGTTTATGTGTTGCTTCTCTGCCGTCATAGCACTATTCAAGGTAATTAAACATGCAATGACTTAATAGTTTTGGCATCATCATACGTATCTTTTAATTTATGTCCTGAACTTTCAGGATATTCCAGATGTTGATGGAGATCGAGACTTTGGTATCCAATCCTTGAGTGTGAGATTGGGGCCTCAAAGAGTAAGAACAGACCCGCATATCTCAGGAAGTGTTTCGAGTAAAACATATGCCTCTGTAAATTGGTACCCCTTTTGAACTTATTTTTTCCTACTGTAGGTGTATCAGCTCTGCATAAGCATATTGTTGACAGCCTATGGCGCTGCCACTCTAGTAGGAGCTTCATCCACAAACCTATTTCAAAAGATCATCACTGTGAGTTATCCGTTGTAAAATCCAGAATTTGAATGAAATACAAACAATTTATAAGCTAAGAAATCTTCCATTGTTGCAATTTTGCATGCTTTGATCATCCAGGTGTCTGGTCATGGCCTGCTTGCTTTGACACTTTGGCAGAGAGCGCAGCACTTTGAGGTTGAAAACCAAGCGCGTGTCACATCATTTTACATGTTCATTTGGAAGGTAATTAATTAAGTTGCTGGCTTATATTGTGCATTCTCTGGACCATTAGACTTTGACTATGTATGCCTAATGATTATTTGCACGTTTTGTGTCACCTTCATGCAGCTATTCTATGCAGAGTATTTCCTTATACCATTTGTGCAGTGA

>HGGT_promoter_region_Falcon

CCAACTGTGCAATTAGGTGTAGAGCATCATGTAGTGGAGGTCGGCGAAGGTGAGGACATGTACAGATGACACATCGACCAGCGCAGGCGCATCGTGATGGAGGAGAGGACGAGGTCACCGATCAGGAGCGGCAGAGGACAAGGTTGAGGAGACGACGCCGCATAGTGGATTCGGCATGGAGGAAGGAGGCTGGCGGCGTTAACCTAGACGCCACGGTGTAGGGCAGCGGGAGTCGATCGGGAAACGCGAGAAGAGCATGGAGGTGGAGGTGTGTTTGTGGCGTGGGTCGTGTGGGCTGGTCTCTCTCTGGAAGGAAGAATTGCCCTGGTTTTCTTAGGGCAGTTTTTTGGTTCGAAGGACACGGGTGTCGGCTCAAAAAAATCGGTGGCTAGCGAGGAGGTCGTGCGAGTAGAGGGGAGCGAACAAGCAAACGAACGACGACATACGGACTCCTCCTTTAGGAGTAGAGAAATGTTAGGAGTGAGATTACGCAACAATCAATTTTGAATCCTAACTTTCCTAACACATGGATTGCCACATAATTGGATTTCATTATTTGATAAGATATTATTGGTTTTCTCCATTTGTAATAATGTGGCATTCAATTTGGAATCCTAACTTTCCTAACACATGGATTGCCACATAATTGGATTTCATTATTTGGTAACGTATTATTGGTTTCCTGCCATTTGTAATTATATGGCAATCAATTTCAAATCCTAACTTTCCTAACACATGGATTGCCACATAAATGGATTTCATTATTTGGTAACGTATTATTAGTTTCCTGCCATTTATAACATGACTGGTTAGAAAAAGTTTGAAAGATTAAGAAAGTTTTTATTGAAAGGTGAAAAAACAAATGTGAAGGCATGGTGGTGCGAGGTGAGACGAAAAAACTGGTTGAAAAAAACCAGTTAAAAAAACCTGGAAGTTATTCTACCATTAGGAGTAGAGATTATCCAAAACTACCAAGGTACTCAGGTAATTTGTCCGACCTCGACTCATTCCACGACCGCAAATCAACCTGTAATTGGATGGTTAGAAGGACAATGGTATCCCCAACCCATCAGGATTCAAGTCCTGGCGTTTACATTATTCCTGGATTTATTTCAGGATTTCAGGTGATACGCTTTTAGTGGGAGGAGACGTTTCCGTCAACCACGAGGCGCCTACGATAACTTTGTAAATCTCAAGATGACATGTCGTCTCAATCTCTATGCTCGTAAGGATAGGGTGTACATGTGTACGTTCGTAGAGATGAATGTATGTGCAGGTATATGGACGTTTGCGCCTGCATAAAAAAAGACCCATTCCACGACCGGGTCGTGACGCACACGCACACCGAAAAAGGATGCATGGCTCGCTCCACCGAGGATCGTGTCGTTTTGGCAAAACGTCTGTGCGTCACACCGGCAGTGAGTCCACCGCACTGCACCATCACCAGCTAGCTAGCGCGCGCGTCCATCTTTTTCTTCAGAATCACATGCCGCCTCCGTCTTTTTCTTTACAATCACACGGCGCATCCGTCTGCATGCAGCCGGATTGCATTACATTACGCACGCCACGCCATGCAGTAATCAATCGGCACATAAACCCCCTCCTCCCCCTGAAGGCCTGAACCTCCCGTCCCATCTGCTCCTCCCCCTCCTTTCACACAGATCCGCGGGTTAACTTCCTCCTCCGGAGGCCGCCCGGCCGGCGAGGATG

>HGGT_promoter_region_Azhul

CCAACTGTGCAATTAGGTGGAGAGCATCATGTAGTGGAGGTCGGCGAAGGTGAGGACATGTACAGATGACACATCGACCAGCGCGGGCGCATCGTGATGGAGGAGAGGACGAGGTCGCCGATCAGGAGCGGCAGAGGACAAGTTTGAGGAGACGACGCCGCATAGTGGATTCGGCATGGAGGAAGGAGGCTGGCGGCGTTAACCTAGACGCCACGGTGTAGGGCAGCGGGAGTCAATCGGGAAACGCAAGAAGAGCATGGAGGTGGAGGTGTGTTTGTGGCGTGGGTCGTGTGGGCTGGTCTCTCTCTGGAAGGAAGAATTGCCCTGGTTTTCTTAGGGCACTTTTTTGGTTTGAAGGACACGGGTGTCGGCTCAAAAAAAATCGGTGGCTAGCGAGGAGGTCGTGCGAGTAGAGGGGAGCGAACAAGCAAACAAACGACGACATACAGACTCCTCCTTTAGGAGTAGAGAAATGTTAGGAGTGAGATTACGCAACAATCAATTTTGAATCCTAACTTTCCTAACACATGGATTGCCACATAATTGGATTTCATTATTTGATAAGGTATTATTGGTTTTCTCCATTTGTAATAATGTGGCATTCAATTTCGAATCCTAACTTTCCTAACACATGGATTGCCACATAATTGGATTTCATTATTTGGTAACGTATTATTGGTTTCCTGCCATTTGTAATTATATGGCAATCAATTTCGAATCCTAACTTTCCTAGCACATGGATTGCCACATAAATGGATTTCATTATTTGGTAAGTATTATTAGTTTCCTGCCATTTGTAACATGACTGGTTAGAAAAAGTTTGAAAGATTAAGAAAGTTTTTATTGAAAGGTGAAAAAACAAATGTGAAGGGATGGTGGTGCGAGGTGAGACGAAAAAACTGGTTGAAAAAAAACAGTTAAAAAAACCTGGAAGTTATTCTACCATTAGGAGTAGAGATTATCCAAAACTACCAAGGTACTCAGGTAATTTGTCCGACCTCGACTCATTCCACGACCGCAAATCAACCTGTAATTGGATGGTTAGAAGGACAATGATATCCCCAGCCCATCAGGATTCAAGTCCTGGTGTTTGCATTATTCCTAGATTTATTTCAGGATTTCGGACGATACGCTTTCAGTAGGAGGAGACTTTTCCGTCAACGACGAGGCGTCTACAATAACTTTGTAAATCTCAAGATGAAATGTCATCTCAGTTTTTCAGAGATGCTTATAAGAATAGGGTGTACATGTATACGTTCATAGAGATGAATGTATGTGCATGTATAAAAACGTTTGCGCCTGCATAAGAAAAAAAACCATTCCACGACCGGGTCGCACACGCACACGCACACGCACACCCGAAAAAGGATGCATGGCTCGCTCCACCGAGGATCGTGTCGTTTTGGCAAGTCATCGACTTTGCCAAAACGTCTGTGCGTCACACCGGCAGTGAGTCCACCGCACTGCACCATCACCAGCTAGCTAGCGCGCGCGTCCATCTTTTTCTTTAGAATCACATGGCGCCTCCGTCTTTTTCTTTACAATCACACGGCGCATCCGCCTGCATGCAGCCGGATTGCATTACATTACGCACGCCACGCCATGCAGTAATCAATCGGCACATAAACCCCCTCCTCCCCCTGAAGGCCTGAACCTCCCGTCCCGTCTGCTCCTCCCCCTCCTTTCACACAGATCCGCGGGTTAACTTCCTCCTCCGGAGGCCGCCCGGCCGGCGAGGATG
