# Supplementary material for: Comparative Systems Biology Reveals Allelic Variation Modulating Tocochromanol Profiles in Barley (Hordeum vulgare L.)
Source: PLoS One. 2014 May 12;9(5):e96276. doi: 10.1371/journal.pone.0096276 (PMC4018352; doi:10.1371/journal.pone.0096276)
Supplement: Table S4 — Falcon x Azhul SNPs coding an amino acid change in HGGT . (DOC) [file pone.0096276.s005.doc]

**Supplementary Table 4** Falcon/Azhul SNPs coding an amino acid change in *HGGT*

| Nucleotide position | Amino acid position  acpoisiont | Falcon | | |  | Azhul | | |
| --- | --- | --- | --- | --- | --- | --- | --- | --- |
|  |  | Nucleotide | Amino acid | Sidechain properties |  | Nucleotide | Amino acid | Sidechain properties |
| 157 | 53 | G | Alanine | Hydrophobic |  | A | Threonine | Polar, neutral |
| 197 | 66 | T | Methionine | Polar, neutral |  | G | Arginine | Basic |
| 278 | 93 | G | Arginine | Basic |  | A | Glutamine | Polar, neutral |
