# Supplementary material for: Comparative Systems Biology Reveals Allelic Variation Modulating Tocochromanol Profiles in Barley (Hordeum vulgare L.)
Source: PLoS One. 2014 May 12;9(5):e96276. doi: 10.1371/journal.pone.0096276 (PMC4018352; doi:10.1371/journal.pone.0096276)
Supplement: Table S5 — Primer sequences used in cloning of gene and promoter regions, SNP genotyping, and quantitative PCR analysis in Falcon, Azhul, and the Falcon x Azhul RIL population. (DOC) [file pone.0096276.s006.doc]

**Supplementary Table 5**  Primer sequences used in cloning of gene and promoter regions, SNP genotyping, and quantitative PCR analysis in Falcon, Azhul, and the FA RIL population

| Function | Target Region | Forward Primer | |  | Reverse Primer | |
| --- | --- | --- | --- | --- | --- | --- |
|  |  | Name | Sequence |  | Name | Sequence |
| Cloning | VTE4 gene | VTE4_F | CAAATACAAAATGGAAAACTCCG |  | VTE4_R | TGTACCTCAATCACATGGGTTC |
|  | VTE4 promoter | VTE4up_F | ACGCTCAAATCCATGAGACA |  | VTE4up_R | TATTTGAAGGGCGAATTCCA |
|  | HGGT gene | HGGT_F | CGCGGGTTAACTTCCTCC |  | HGGT_R | GCCCTTGTACAAATTTCACTGC |
|  | HGGT promoter | HGGTup_F | CCAACTGTGCAATTAGGTGGA |  | HGGTup_R_Falcon  HGGTup_R_Azhul | AGCTTGCGAAATGGCACTGA  AGCAGCTTGAGAAATGGCACTG |
| HRMA ^a^ | VTE4 SNP | VTE4_HRM_F | GAGTCACTGCAAAACTGTTCAC |  | VTE4_HRM_R | TATATATCGACCCAGTCTGTCACACAA |
|  | HGGT SNP | HGGT_HRM_F | GGATATTCCAGATGTTGATGGA |  | HGGT_HRM_R | CACTCAAGGATTGGATACCAAAG |
| qPCR ^b^ | VTE4 | VTE4_qPCR_F | CTGAAACCTGACGAGCTGAA |  | VTE4_qPCR_R | CCACGTTTTCAGACCAGTCA |
|  | HGGT | HGGT_qPCR_F | TGATTATCCAGCCCTCCAAG |  | HGGT_qPCR_R | TGTGGCCTGCAAAATTCATA |

^a^ High-resolution melt analysis (HRMA) used for SNP genotyping

^b^ quantitative PCR used for gene expression analysis
